# Mechanistic insights into hydroxy(tosyloxy)iodobenzene-mediated ditosyloxylation of chalcones: a DFT study

**DOI:** 10.3762/bjoc.21.208

**Published:** 2025-12-16

**Authors:** Jai Parkash, Sangeeta Saini, Vaishali Saini, Omkar Bains, Raj Kamal

**Affiliations:** 1 Department of Chemistry, Kurukshetra University, Kurukshetra, Haryana 136119, India; 2 School of Basic and Applied Sciences, Raffles University, Neemrana, Rajasthan 301705, India

**Keywords:** 1,2-aryl-migration, chalcones, ditosyloxylation, HTIB, *para*-substituents, α,β-unsaturated carbonyl compounds

## Abstract

In this paper, the mechanism of the hydroxy(tosyloxy)iodobenzene (HTIB)-mediated conversion of chalcones (α,β-unsaturated carbonyl compounds) to ditosyloxy ketones is investigated. Here, at β-carbon of the chalcone, an aryl group with a *para*-substituent is present. Our study focuses on investigating the effect of different nature of *para*-substituents on the reaction mechanism. The substituents considered in the study include -OCH_3_, -SCH_3_, -Cl and -NO_2_ groups. For these chalcones, different possible pathways at various steps during the reaction are investigated leading to formation of α,β-ditosyloxy ketones and β,β-ditosyloxy ketones. It is found that the mechanism for the formation of α,β-ditosyloxy ketone involves only electrophilic addition of HTIB, and the mechanism is the same for all studied chalcones, irrespective of whether an electron-donating or electron-withdrawing substituent is present on the aryl ring. However, the detailed mechanism for the formation of β,β-ditosyloxy ketones is different and depends on the nature of the substituent. Broadly, the formation of β,β-ditosyloxy ketones involves electrophilic addition followed by 1,2-aryl migration. Our study shows that the presence of an electron-donating group on the migrating aryl ring favours the formation of β,β-ditosyloxy ketones while in case of electron-withdrawing groups, there are nearly equal chances of the formation of α,β-ditosyloxy ketones and β,β-ditosyloxy ketones.

## Introduction

Hypervalent iodine compounds exhibit a range of bonding patterns which making these compounds powerful reagents or catalysts for a number of organic transformations [1–4] – oxidation of alcohols [5], epoxidation of alkenes [6], oxidative dearomatization [7], amination [8], oxidative coupling [9], ring contraction [10–12], oxidative rearrangement [13–15], dihydroxylation of alkenes, arylation [16], oxidation of sulfides [17] and many more. These hypervalent compounds contain iodine in higher oxidation state than its usual −(I) valence. The reactivity characteristics of these compounds are similar to transition metal reagents, as a result these hypervalent compounds can be used as an alternative. For example, iodobenzene diacetate (PhI(OAc)_2_), can be used to selectively oxidize alcohols to aldehydes or ketones over toxic chromium(VI) compounds [18–19]. Hypervalent iodine(III) reagents have synthetic importance not only due to their nontoxic nature, but also because of milder reaction conditions, higher atom economy, and multipurpose oxidative characteristics over toxic/expensive heavy metal reagents containing Tl(III) or Pb(IV). Due to their versatility, eco-friendliness and low cost, the organo hypervalent iodine reagents have become popular in the field of synthetic chemistry in the last few decades.

Hydroxy(tosyloxy)iodobenzene (HTIB) is a hypervalent iodine(III) reagent that contains both a hydroxy and a tosyloxy group. Oxidative rearrangement is one of the important reactions induced by HTIB. Similar to other hypervalent iodine(III) compounds, HTIB is known to have higher electrophilicity towards olefinic bonds. HTIB dissociates, adds onto the olefinic bond and subsequently acts as a good leaving group resulting in generation of a cationic intermediate which facilitates 1,2-aryl migration [20–23]. Similar types of oxidative rearrangements in α,β-unsaturated diaryl ketones that leads to the α-aryl-β,β dioxygenated skeleton via 1,2-aryl migration have been studied by using different reagents such as Tl(OCOCH_3_)_3_/CH_3_OH, Tl(ONO_2_)_3_/CH_3_OH, PhI(OCOCH_3_)_2_/CH_3_OH and PhI(OH)OTs/CH_3_OH in polar nucleophilic solvents [24–28].

A number of experimental and computational studies have explored the reaction pathway for these oxidative rearrangement reactions under metal-free conditions with the use of hypervalent iodine(III) compounds in polar nucleophilic or non-nucleophilic solvents [10–12,20,29–36]. Fujita discusses typical pathways of alkene oxidation with hypervalent iodine in great detail including the stereochemical course of reaction involving a cyclic iodonium ion [30]. In the literature, however, a number of studies also report the formation of a carbenium ion as an alternate pathway, typically for the oxidation of alkenes with phenyl substituents on carbon–carbon double bonds [10–12,29,34–35].

**Scheme 1:**
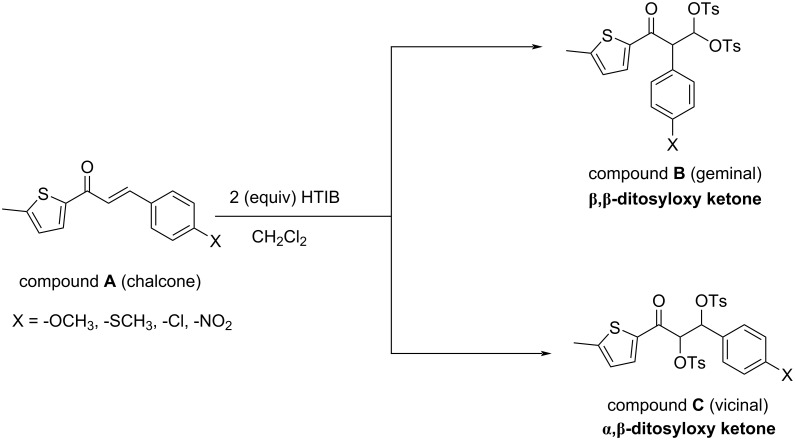
Structure of reactant (chalcone, compound **A**), geminal product (compound **B**), vicinal product (compound **C**), and the different substituents on aryl ring (denoted by X) considered in the current study.

Here, Scheme 1 depicts the reaction investigated in the current study which is, HTIB-mediated ditosyloxylation of α,β-unsaturated carbonyl compounds (chalcones) bearing an aryl group at β-position (compound **A**) leading to formation of two possible products, that are: α-arylated β,β-ditosyloxy-substituted carbonyl compound (compound **B** – geminal product) and β-arylated α,β-ditosyloxy-substituted carbonyl compound (compound **C** – vicinal product). By systematically varying the substituent (-X) on *para*-position of the aryl group, the influence of different electron-donating and electron-withdrawing groups on the reaction mechanism of these modified chalcone compounds is studied computationally. The results obtained for chalcones are in qualitative agreement with results reported for substituted styrenes [31].

## Computational Methodology

Quantum chemical calculations were carried out using Gaussian 09 package [37]. The hybrid exchange-correlation functional B3LYP-D3 (including dispersion) was used. B3LYP combines the Becke’s three parameter exchange functional, and the Lee–Yang–Parr correlation functional [38–39]. The D3 correction accounts for dispersion interactions, which are important for systems with heavy atoms [40–41]. Mixed basis set LANL2DZ (d, p) was employed for iodine while the 6-31G+(d,p) was used for remaining other atoms. The geometries of all ground state and transition state structures are optimized using the above specified basis sets and exchange-correlation functional. The effect of solvent was also taken into account using the SMD implicit solvation model. The solvent considered for the present study was dichloromethane (CH_2_Cl_2_). Frequency calculations were performed for all optimized structures to confirm the stationary point and transition state. Intrinsic reaction coordinate (IRC) calculations were carried out to validate the transition states. The reported energies are given in kcal/mol and bond lengths are reported in Å.

## Results and Discussion

In this section, firstly the mechanistic details of the pathway leading to the formation of β,β-ditosyloxy ketones are discussed followed by the same for the formation of α,β-ditosyloxy ketones. Finally, the results for the two pathways are compared to draw the inferences. The main focus of the current study is to understand the addition mechanism and the migratory aptitude of the aryl ring (present at β-position of reactant – chalcone) depending on the nature of the substituent present at *para*-position. The *para*-substituents studied are -OCH_3_, -SCH_3_, -Cl and -NO_2_. The electron-donating groups are -OCH_3_ and -SCH_3_, while the remaining two are electron-withdrawing groups with relative power of -Cl < -NO_2_. Note, the formation of β,β-ditosyloxy ketone from chalcone (Scheme 1) with X = -OCH_3_ is already discussed in reference [34]. Therefore, here for X = -OCH_3_ only those results for β,β-ditosyloxy ketone formation are presented which are required for comparison with the other *para*-substituents.

### Formation of β,β-ditosyloxy ketones

**Scheme 2:**
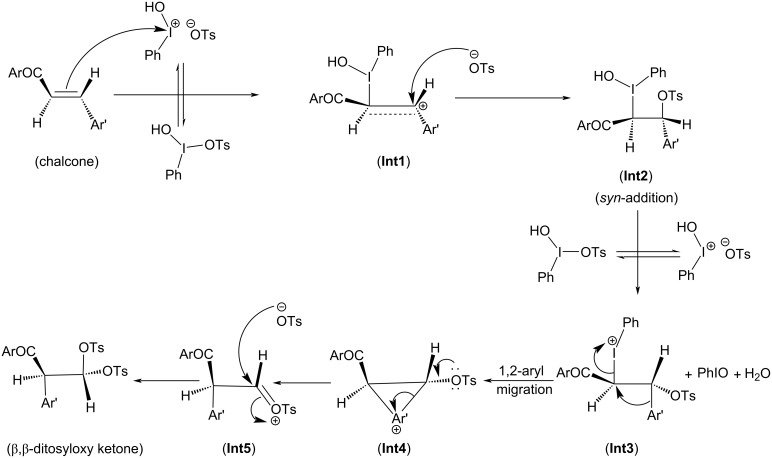
Reaction mechanism of ditosyloxylation of chalcones with X = -OCH_3_ , -SCH_3_ followed by 1,2-aryl migration leading to the formation of β,β-ditosyloxy ketones.

**Scheme 3:**
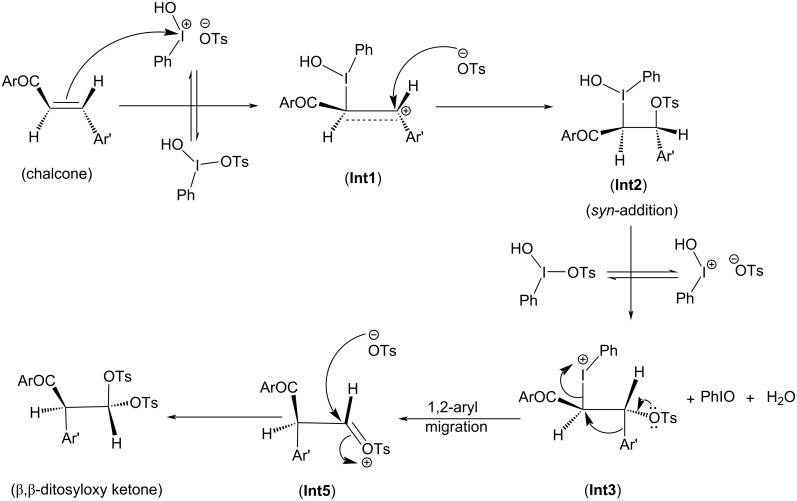
Reaction mechanism of ditosyloxylation of chalcones with X = -Cl, -NO_2_ leading to the formation of β,β-ditosyloxy ketones.

**Figure 1:**
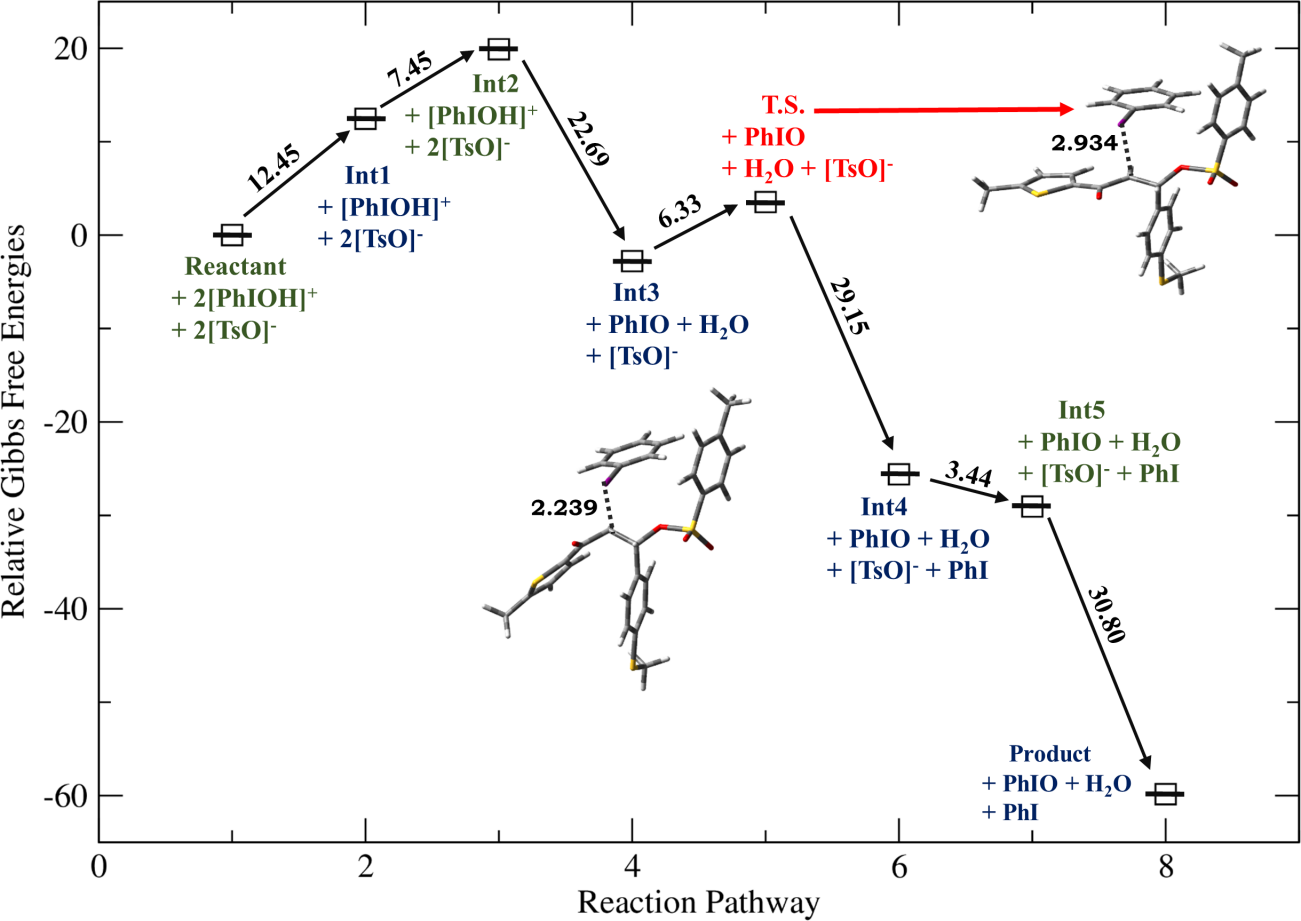
Relative Gibbs free energy profile for HTIB-mediated ditosyloxylation of chalcone with X = -SCH_3_ involving 1,2-aryl migration leading to the formation of the β,β-ditosyloxy ketone. Free energies are reported in kcal/mol. Bond lengths are reported in Å.

**Figure 2:**
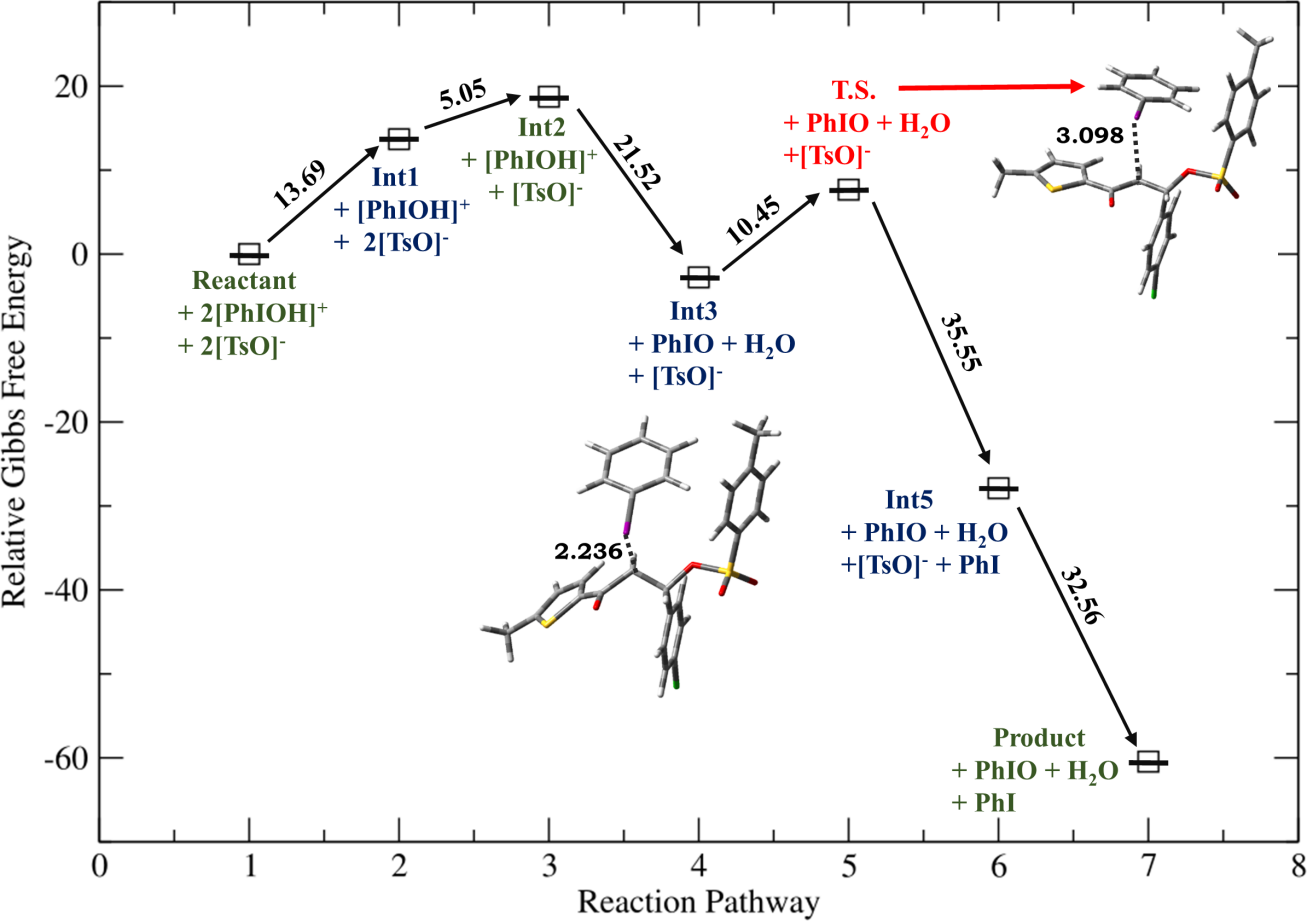
Relative Gibbs free energy profile for HTIB-mediated ditosyloxylation of chalcone with X = -Cl involving 1,2-aryl migration leading to the formation of the β,β-ditosyloxy ketone. Free energies are reported in kcal/mol. Bond lengths are reported in Å.

**Figure 3:**
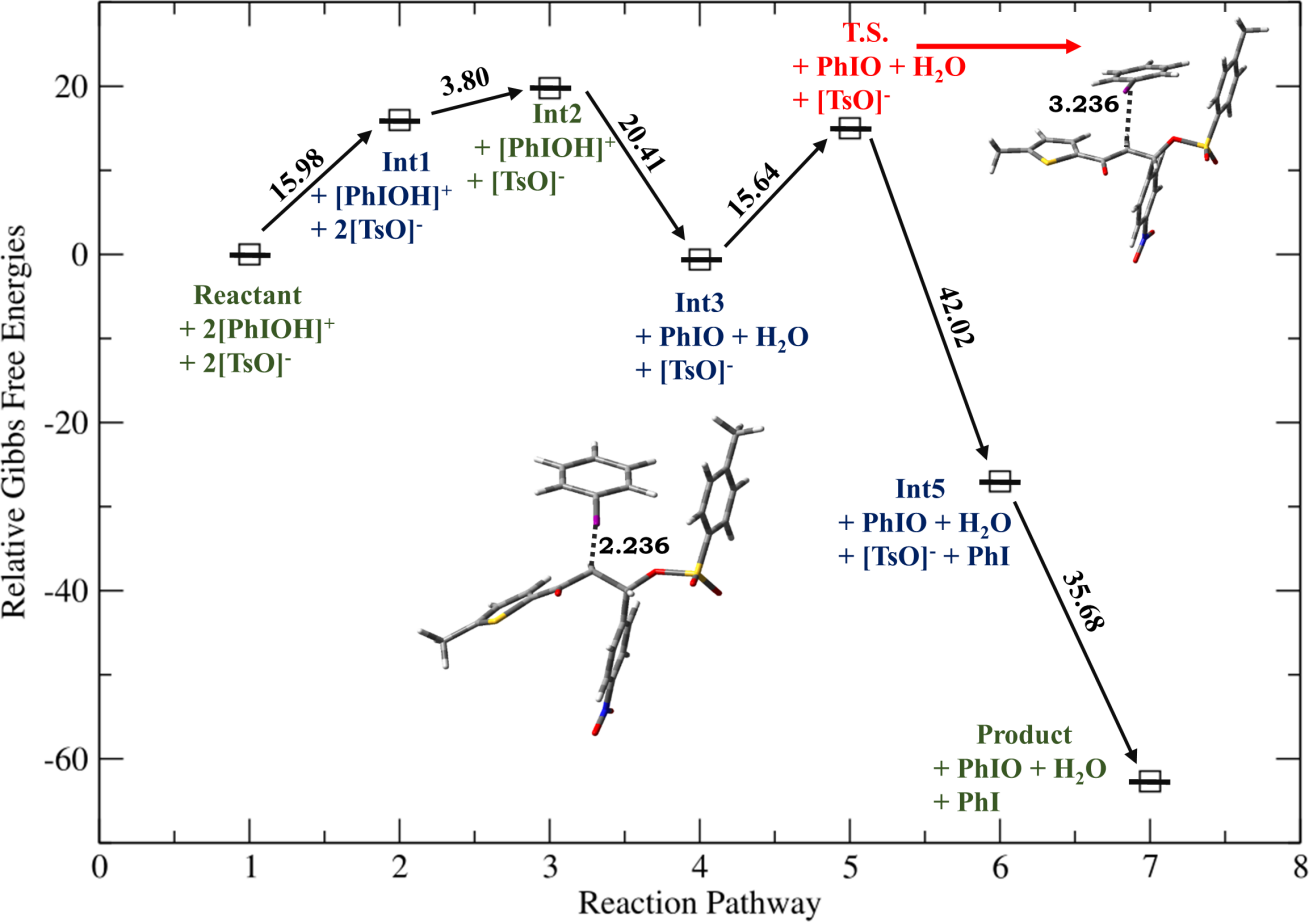
Relative Gibbs free energy profile for HTIB-mediated ditosyloxylation of chalcone with X = -NO_2_ involving 1,2-aryl migration leading to the formation of the β,β-ditosyloxy ketone. Free energies are reported in kcal/mol. Bond lengths are reported in Å.

Depending on the nature of the *para*-substituent present on the aryl ring, the detailed calculated reaction mechanism differs. Scheme 2 and Scheme 3 depicts the reaction mechanism for these two possible cases, i.e., when X is electron-donating and electron-withdrawing, respectively. Figure 1 depicts the free energy reaction profile for the chalcone with X = -SCH_3_. The corresponding reaction profile for X = -OCH_3_ has been discussed in our earlier study [34]. Figure 2 and Figure 3 present the free energy reaction profiles for chalcones with X = -Cl and X = -NO_2_, respectively. Table 1 lists the free energy and potential energy for various intermediates and transition states for chalcones with different substituents, X = -SCH_3_, -Cl, -NO_2_ including the data for X = -OCH_3_. The difference between free energies for consecutive reaction species are shown over arrows in Figures 1, 2 and 3 for substituents X = -SCH_3_, -Cl and -NO_2_, respectively. Table 1 also reports these free energy differences as given in parenthesis for all studied substituents including -OCH_3_.

**Table 1:** Effect of substituent group present on the migrating aryl ring on relative free energies (*G*) and relative potential energies (*E*) of the transition state, intermediates and product for the reaction pathway leading to formation of β,β-ditosyloxy ketones. The energies are reported relative to the reactant corresponding to each substituent. The values in parenthesis are the free energy differences between consecutive reaction species. The difference in potential energy of **Int2** and **Int1** for substituted chalcones is underlined and shown in bold. Energies are reported in kcal/mol.

B3LYP-D3/6-31G +(d,p)/LANL2DZ(d,p)								

-OCH_3_ group as a substituent								

Energy	Reactant	**Int1**	**Int2**	**Int3**	**T.S.**	**Int4**	**Int5**	Product

*G*	0	11.12	18.61 **(+7.49)**	−1.22 **(−19.83)**	1.80 **(+3.02)**	−26.44 **(−28.24)**	−29.27 **(−2.83)**	−60.04 **(−30.77)**
*E*	0	−2.64	−7.50 **−4.86**	−20.68	−15.54	−30.91	−32.64	−77.57

-SCH_3_ group as a substituent								

*G*	0	12.45	19.90 **(+7.45)**	−2.79 **(−22.69)**	3.54 **(+6.33)**	−25.61 **(−29.15)**	−29.05 **(−3.44)**	−59.85 **(−30.80)**
*E*	0	−1.39	−6.47 **−5.08**	−19.85	−13.90	−29.08	−32.41	−77.90

-Cl group as a substituent								

*G*	0	13.69	18.74 **(+5.05)**	−2.78 **(−21.52)**	7.67 **(+10.45)**	–	−27.88 **(−35.55)**	−60.44 **(−32.56)**
*E*	0	0.39	−7.71 **-8.10**	−20.01	−9.16	–	−31.15	−78.43

-NO_2_ group as a substituent								

*G*	0	15.98	19.78 **(+3.80)**	−0.63 **(−20.41)**	15.01 **(+15.64)**	–	−27.01 **(−42.02)**	−62.69 **(−35.68)**
*E*	0	2.38	−7.53 **−9.91**	−18.09	−2.14	–	−30.02	−80.77

Overall both in Scheme 2 and Scheme 3, the reaction between chalcone and HTIB proceeds through electrophilic addition of [PhIOH]^+^ followed by nucleophilic addition of ^−^OTs. In the beginning electrophilic addition occurs on the chalcone molecule resulting in the formation of carbenium ion **Int1**. The positive charge is better accommodated at β-position because of the presence of the aryl group with X = -OCH_3_, -SCH_3_ compared to the α-position which is adjacent to the carbonyl group. The analysis of data corresponding to **Int1** as presented in Table 1 confirms that electron-donating groups stabilize **Int1** more than electron-withdrawing groups, as expected. The electrophilic addition of [PhIOH]^+^ is driven by the electron-rich double bond present in the chalcone structure. Therefore, in case of chalcones with electron-withdrawing groups, the interaction of [PhIOH]^+^ with C at α-position is weaker and is evident by longer C–I bond lengths in **Int1**, i.e., 3.06 Å and 3.14 Å for chalcones with X = -Cl, -NO_2_, respectively (Table 2). In fact, this interaction is much weaker for chalcone with X = -NO_2_ that the reaction between chalcone and HTIB leading to formation of ditosyloxy ketone may not occur at all [42].

**Table 2:** For the reaction pathway leading to the formation of β,β-ditosyloxy ketones – calculated bond lengths (Å) between atoms in intermediate **1** and **2** (**Int1** and **Int2**) with different substituent group (X = -OCH_3_, -SCH_3_, -Cl and -NO_2_) present at *para*-position on the migrating aryl group.

Intermediate	O–H	I–OH	C–I

**Int1** (-OCH_3_)	0.97	2.02	2.93
**Int2** (-OCH_3_)	0.97	2.20	2.35
**Int1** (-SCH_3_)	0.97	2.02	2.95
**Int2** (-SCH_3_)	0.97	2.20	2.35
**Int1** (-Cl)	0.97	2.00	3.06
**Int2** (-Cl)	0.97	2.20	2.35
**Int1** (-NO_2_)	0.97	1.99	3.14
**Int2** (-NO_2_)	0.97	2.19	2.36

The subsequent nucleophilic addition of ^−^OTs on **Int1** takes place on β-position specifically in a *syn*-manner with respect to the carbon–iodine bond. This nucleophilic *syn-*addition leads to the formation of an intermediate referred here as **Int2**. The *syn*-addition refers to the direction of approach of the nucleophile, i.e., the tosylate ion from the same side as the carbon–iodine bond. In our earlier study on the oxidative rearrangement mediated by HTIB in chalcone – (*E*)-3-(4-X-phenyl)-1-(5-methylthiophen-2-yl)prop-2-en-1-one (Scheme 1, Compound A, X = -OCH_3_), the nucleophilic addition of the tosylate to carbenium ion is found to be *syn*. An *anti*-addition was also studied but it is found to be an unfavourable conformation for subsequent aryl migration [34]. On account of this, the current study focuses on the *syn*-addition mechanism.

The presence of a positive charge at the β-position in **Int1** is unfavourable for compounds bearing an electron-withdrawing group on the phenyl ring (present at β-position). As a result, speaking in terms of potential energy **Int2** (in reference to **Int1**) is more stabilized for chalcones with X = -Cl, -NO_2_ over chalcones with X = -OCH_3_, -SCH_3_ (Table 1). For reference the difference in potential energy of **Int2** and **Int1** for substituted chalcones is underlined and shown in bold (Table 1). A significant change in the bond length of I–OH in **Int2** is observed. Table 2 reports bond lengths related to the interaction between chalcone and [PhIOH]^+^ for **Int1** and **Int2**. The significant lengthening of the I–OH bond facilitates removal of the -OH group from **Int2** to form **Int3**. The dissociation of I–OH is an important step in the reaction mechanism which plays a significant role in the overall reaction. The chemical transformation of **Int2** to **Int3** involves the interaction between lengthened the I–OH bond with [PhIOH]^+^ furnished by the second dissociated molecule of HTIB. This interaction with subsequent removal of H_2_O results in the formation of iodosobenzene. The formation of iodosobenzene makes the transformation of **Int2** to **Int3** thermodynamically favourable. The supporting data is provided in Table S1 in Supporting Information File 1.

The chemical transformation of **Int3** to **Int5** involves significant difference in the reaction mechanism depending upon whether X is electron-donating or electron-withdrawing. For X = -OCH_3_, -SCH_3_ a three-membered cyclic carbocation (**Int4**) is involved. This distinction can be attributed to the substituent on the phenyl ring. The positive charge on the three-membered cyclic carbocation will be stabilized by an electron-donating substituent (X) while the electron-withdrawing groups, X = -Cl, -NO_2_ would destabilize such an intermediate. Therefore, for chalcones with X = -Cl, -NO_2_ intermediate **Int4** does not form. Hence, for chalcones with X = -OCH_3_, -SCH_3_ the reaction profile is:

Reactants **→ Int1 → Int2 → Int3 → T.S. → Int4 → Int5 →** β,β-ditosyloxy ketone (geminal product).

while the reaction profile of chalcones with X = -Cl, -NO_2_ is:

Reactants **→ Int1 → Int2 → Int3 → T.S. → Int5 →** β,β-ditosyloxy ketone (geminal product).

In **Int5**, the phenyl ring is present at α-position. The tosyloxy group present at β-position facilitates the migration of aryl ring as shown in Scheme 2 (**Int4**) and Scheme 3 (**Int3**).

### Formation of α,β-ditosyloxy ketones

**Scheme 4:**
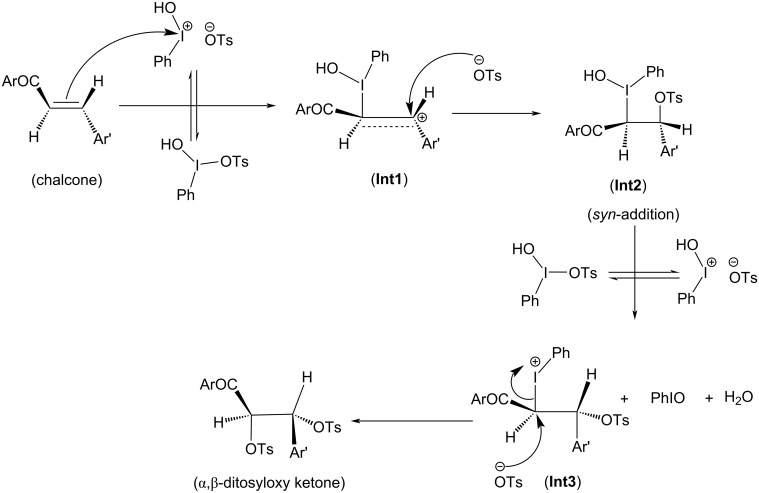
Reaction mechanism of ditosyloxylation of chalcones with X = -OCH_3_, -SCH_3_, -Cl, -NO_2_ leading to the formation of α,β-ditosyloxy ketones. Herein, the tosylate ion attacks at α-carbon position of **Int3** leading to the difference.

It is also important to mention here that for the liberation of IPh from **Int3**, two possible paths exist. One that involves aryl migration and leads to the formation of β,β-ditosyloxy ketones (geminal product) as shown in Scheme 2 and Scheme 3. The other path involves an S_N_2 type attack of the tosylate ion on the α-carbon resulting in the formation of α,β-ditosyloxy ketones (vicinal product) (Scheme 4). Here, no aryl migration takes place. In Scheme 4, the reaction profile is:

Reactants **→ Int1 → Int2 → Int3 → T.S. →** α,β-ditosyloxy ketone (vicinal product).

**Figure 4:**
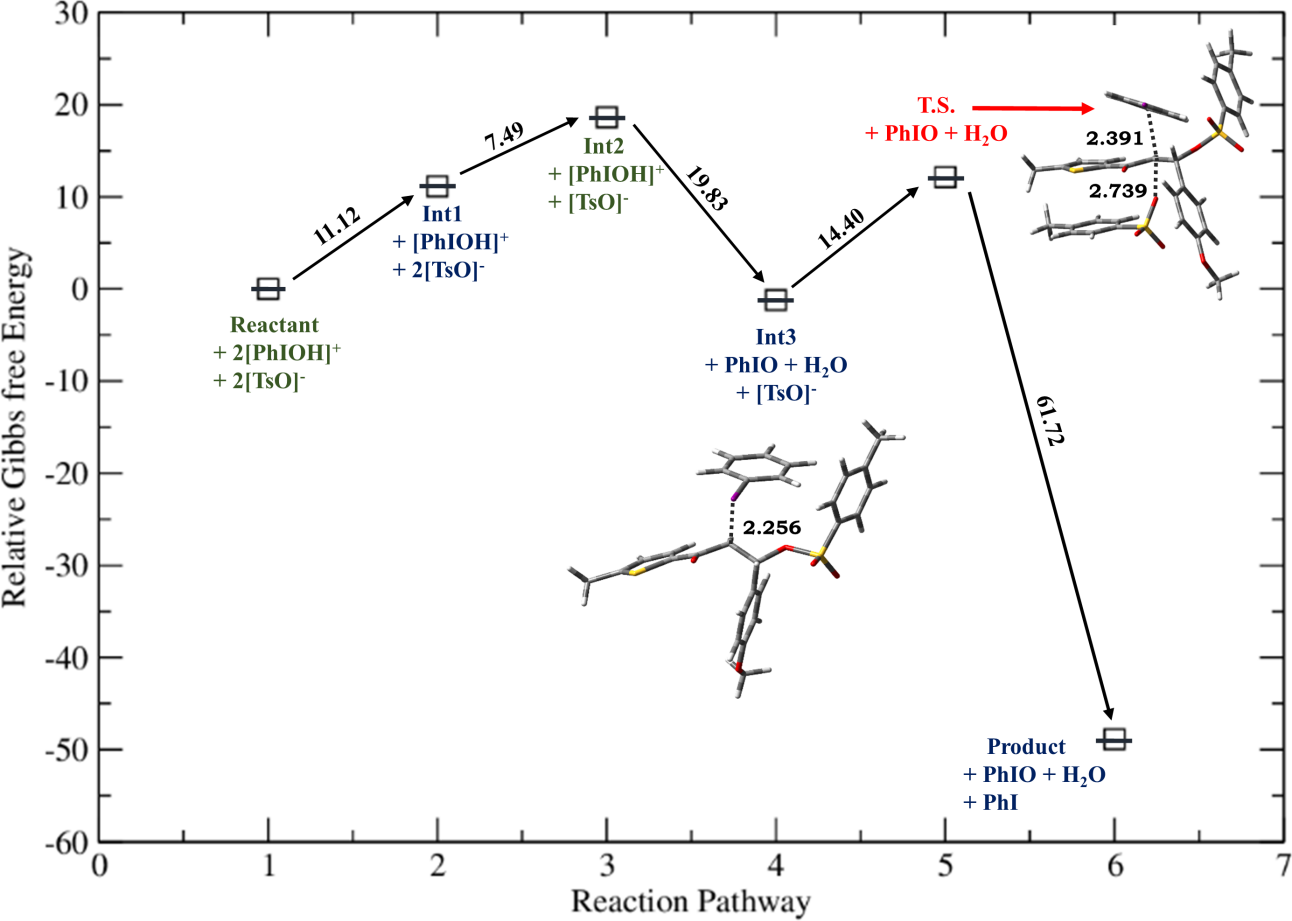
Relative Gibbs free energy profile for HTIB-mediated ditosyloxylation of chalcone with X = -OCH_3_ leading to the formation of the α,β-ditosyloxy ketone. Free energies are reported in kcal/mol. Bond lengths are reported in Å.

**Figure 5:**
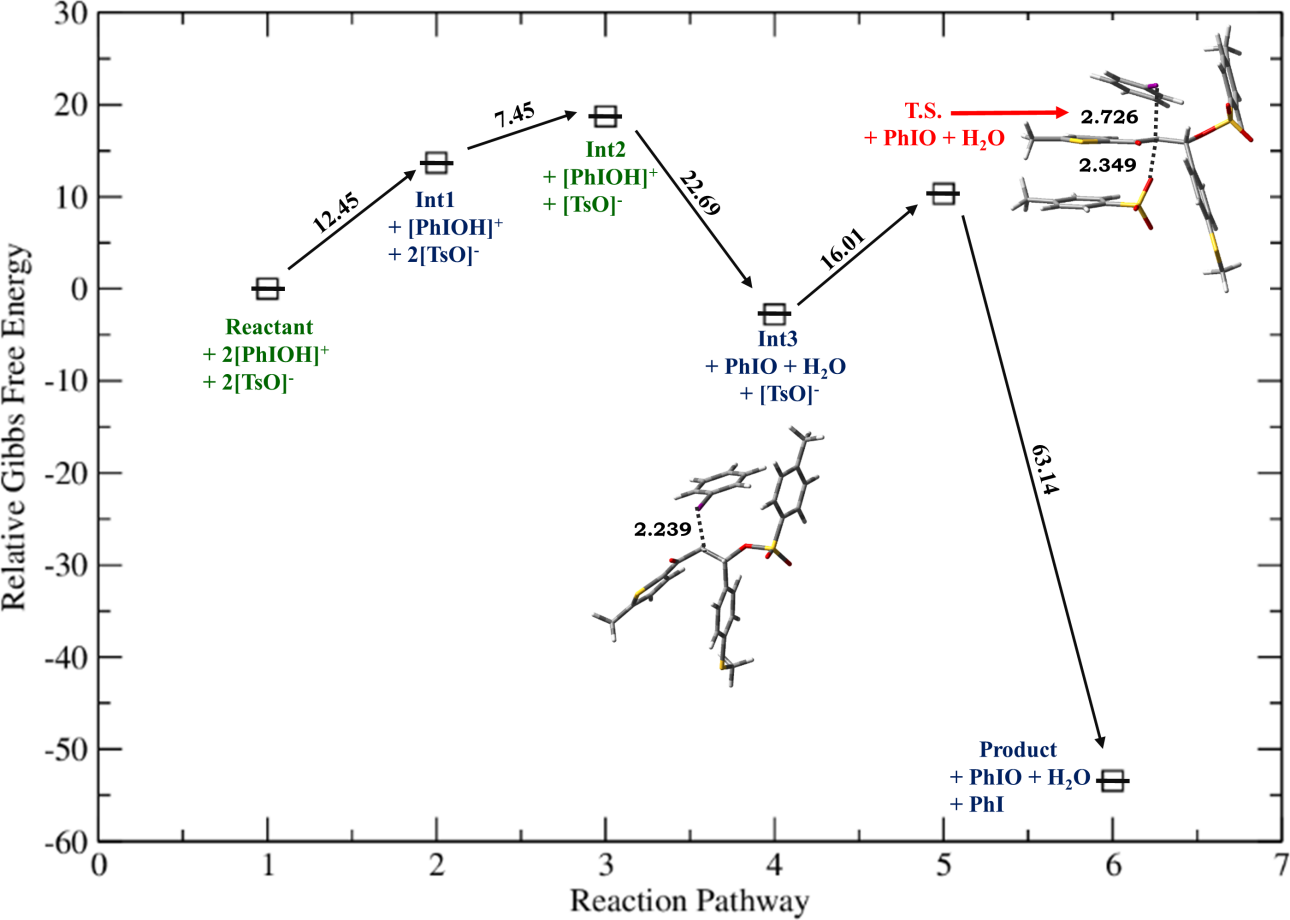
Relative Gibbs free energy profile for HTIB-mediated ditosyloxylation of chalcone with X = -SCH_3_ leading to the formation of the α,β-ditosyloxy ketone. Free energies are reported in kcal/mol. Bond lengths are reported in Å.

**Figure 6:**
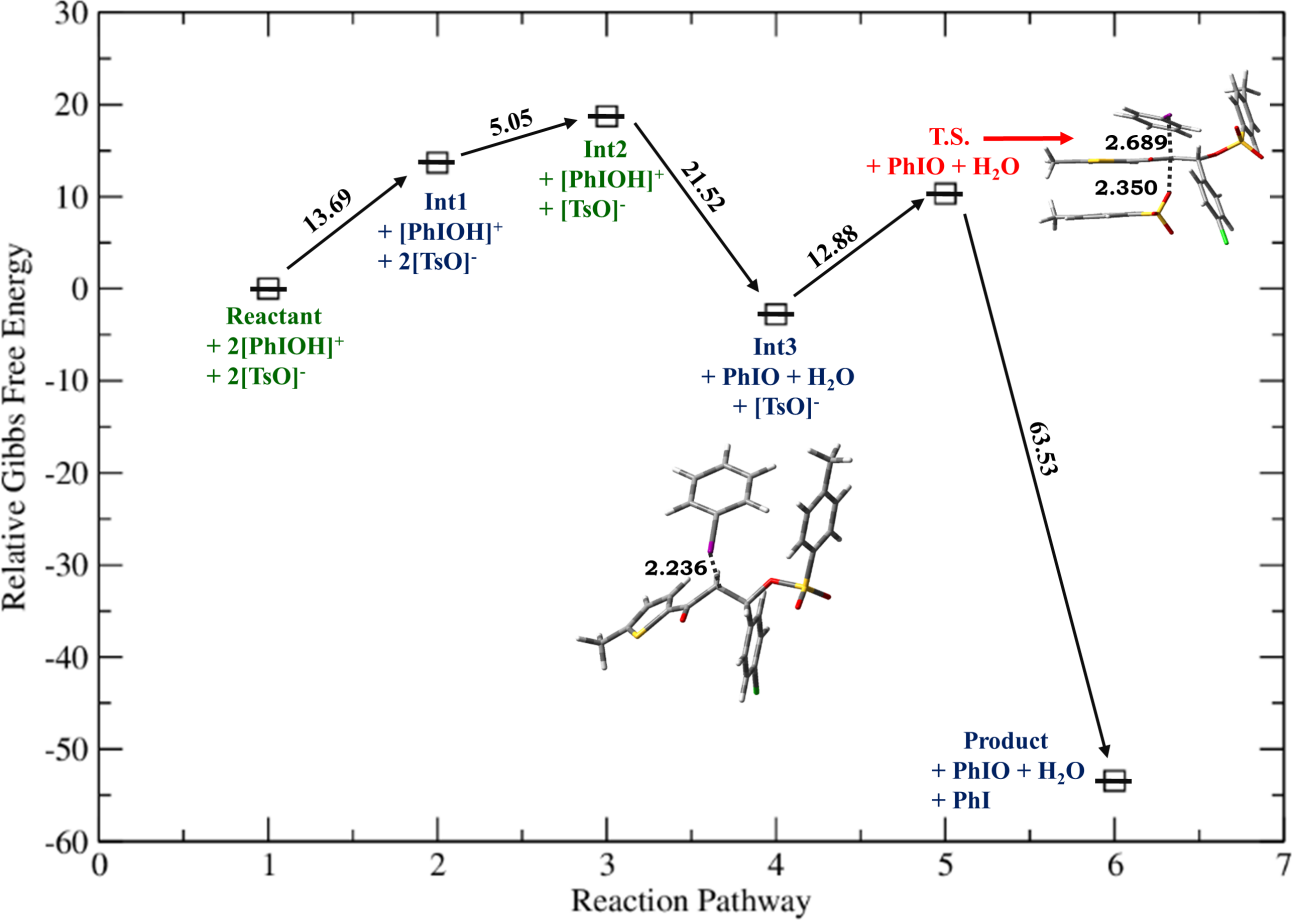
Relative Gibbs free energy profile for HTIB-mediated ditosyloxylation of chalcone with X = -Cl leading to the formation of the α,β-ditosyloxy ketone. Free energies are reported in kcal/mol. Bond lengths are reported in Å.

**Figure 7:**
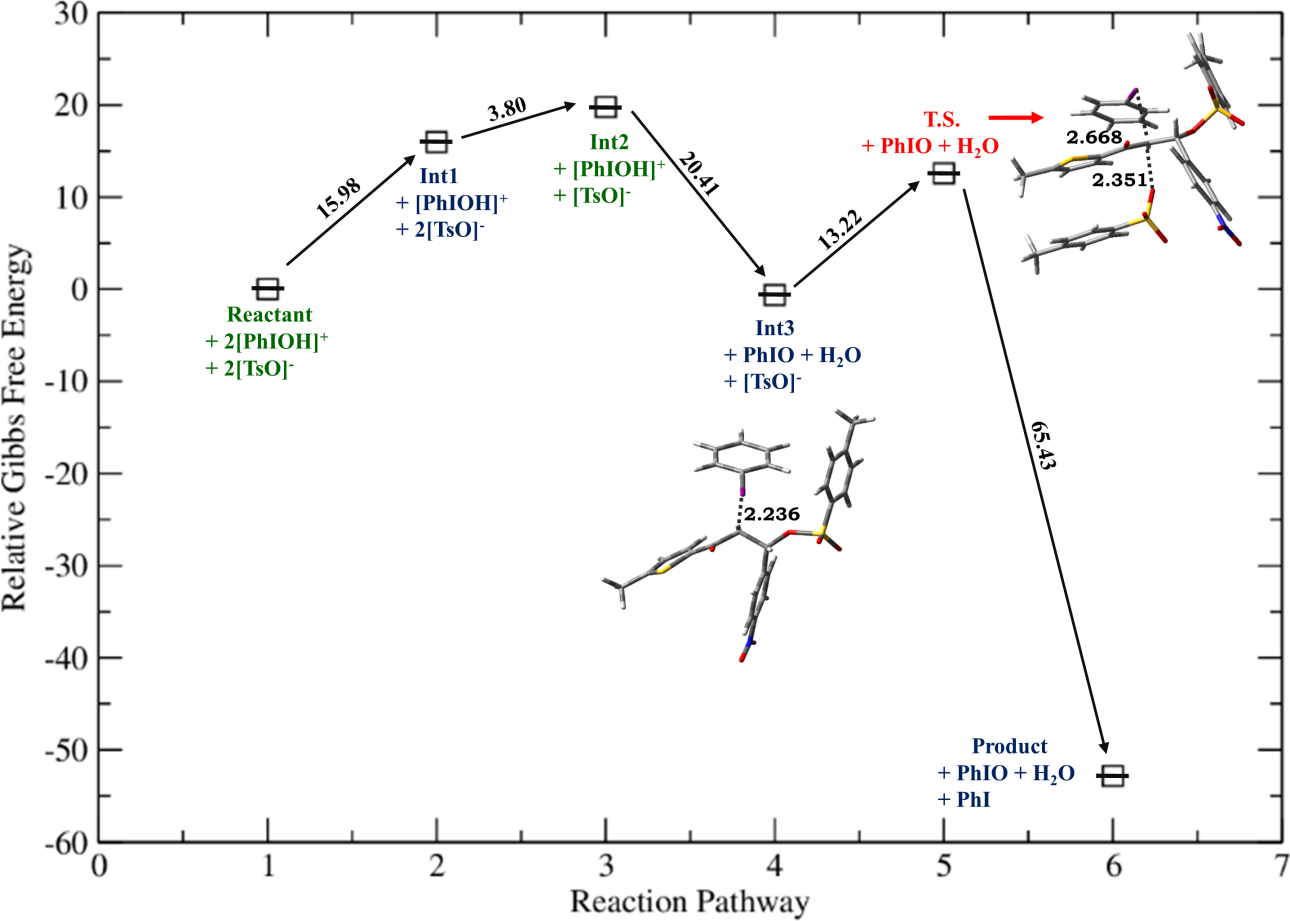
Relative Gibbs free energy profile for HTIB-mediated ditosyloxylation of chalcone with X = -NO_2_ leading to the formation of the α,β-ditosyloxy ketone. Free energies are reported in kcal/mol. Bond lengths are reported in Å.

These two pathways are further elaborated below and Table 3 lists the free energies and potential energies for various intermediates and transition states for chalcones with different X = -OCH_3_, -SCH_3_, -Cl, -NO_2_ for the reaction proceeding as per Scheme 4 leading to formation of α,β-ditosyloxy ketones. Here, the difference between free energies for the consecutive reaction species are shown over arrows in Figures 4–7 for substituents X = -OCH_3_, -SCH_3_, -Cl and -NO_2_, respectively. Table 3 also reports these free energy differences as given in parenthesis for all studied substituents.

**Table 3:** Effect of the substituent group present on the aryl ring on relative free energies (*G*) and relative potential energies (*E*) of the transition states, intermediates and products for the reaction pathway leading to the formation of α,β-ditosyloxy ketones (Scheme 4). The energies are reported relative to the reactant corresponding to each substituent. The values in parenthesis are the free energy differences between the consecutive reaction species. Energies are reported in kcal/mol.

Energy	Reactant	**Int1**	**Int2**	**Int3**	Transition state	Product

–OCH_3_						

*G*	0	11.12	18.61 **(+7.49)**	−1.22 **(−19.83)**	13.18 **(+14.40)**	−48.54 **(−61.72)**
*E*	0	−2.64	−7.50	−20.68	−20.19	−66.89

–SCH_3_						

*G*	0	12.45	19.90 **(+7.45)**	−2.79 **(−22.69)**	13.22 **(+16.01)**	−49.92 **(−63.14)**
*E*	0	−1.39	−6.47	−19.85	−20.03	−69.53

–Cl						

*G*	0	13.69	18.74 **(+5.05)**	−2.78 **(−21.52)**	10.10 **(+12.88)**	−53.43 **(−63.53)**
*E*	0	0.39	−7.71	−20.01	−20.90	−72.25

–NO_2_						

*G*	0	15.98	19.78 **(+3.80)**	−0.63 **(−20.41)**	12.59 **(+13.22)**	−52.84 **(−65.43)**
*E*	0	2.38	−7.53	−18.09	−20.66	−72.76

### Comparison of two pathways

In **Int3** with the removal of the hydroxy group, a positive charge is present on the iodine atom. As discussed earlier, for X = -OCH_3_, -SCH_3_ the presence of electron-donating groups in the aryl moiety favours the concerted removal of IPh and attack of the aryl group at the α-position which leads to the formation and stabilization of the three-membered cyclic carbocation (**Int4**). The electron-donating group provides electron density to the carbocation, which stabilizes the positive charge. As a result, **Int4** is observed when electron-donating groups are present (Scheme 2). In **Int4**, the tosyloxy group at β-position donates electrons to complete the 1,2-aryl migration and gives **Int5**. Subsequently, the positive charge at the β-carbon (**Int5**) makes it prone to nucleophilic attack by another tosylate ion and β,β-ditosyloxy ketones (geminal) are obtained as the products.

For X = -Cl, -NO_2_; the DFT study shows that the three-membered cyclic carbocation is not formed (Scheme 3). Therefore, to obtain β,β-ditosyloxy ketones as products from such chalcones, the possible mechanism is that, in **Int3** the tosyloxy group at β-position donates electrons and the negatively charged aryl group is released which attacks at α-position to give **Int5** and subsequently upon attack by another tosylate ion the β,β-ditosyloxy ketone (geminal product) is obtained.

Now, for compounds with X as electron-withdrawing group, there exist the equal possibility that the tosylate ion from the second molecule of dissociated HTIB attacks at the α-position resulting in release of IPh from **Int3**. This attack would lead to the formation of α,β-ditosyloxy ketones (vicinal product) (Scheme 4). It is evident from the comparison between reaction profiles shown in Figure 6 and Figure 7 vis-à-vis reaction profile shown in Figure 2 and Figure 3. For chalcones with X = –Cl, –NO_2_ the activation Gibbs free energies for step **Int3 → T.S.** → vicinal product (Scheme 4) for the formation of the α,β-ditosyloxy ketones are 12.88 kcal/mol, 13.22 kcal/mol, respectively, while for the formation of the β,β-ditosyloxy ketones, the corresponding barriers for step **Int3 → T.S. → Int5** (Scheme 3) are 10.45 kcal/mol, 15.64 kcal/mol, respectively. The energy costs of overcoming these barriers and formation of α,β-ditosyloxy ketones or β,β-ditosyloxy ketones are nearly the same. Therefore, both vicinal and geminal products may equally be likely. But the same is not true when X is an electron-donating group.

For X = -OCH_3_, -SCH_3_ activation Gibbs free energies for step **Int3 → T.S. →** vicinal product (Scheme 4) leading to formation of α,β-ditosyloxy ketones are 14.40 kcal/mol, 16.01 kcal/mol, respectively, while in case of the formation of β,β-ditosyloxy ketones (Scheme 2), the corresponding barriers for step **Int3 → T.S. → Int4** are 3.02 kcal/mol, 6.33 kcal/mol (see Figure 4, Figure 5 vis-à-vis Table 1, Figure 1), respectively. From the comparative analysis of free energy barriers involved in the formation of β,β-ditosyloxy ketone and α,β-ditosyloxy ketone (as tabulated in Table 1 and Table 3, respectively) it can be concluded that in case of chalcones with electron-donating substituent, the formation of β,β-ditosyloxy ketone is energetically more favourable over the formation of α,β-ditosyloxy ketone.

## Conclusion

The propensity of 1,2-aryl migration in chalcone – (*E*)-3-(4-X-phenyl)-1-(5-methylthiophen-2-yl)prop-2-en-1-one with X = -OCH_3_, -SCH_3_, -Cl, -NO_2_ in presence of HTIB under non-aqueous conditions is studied. The current study highlights the differences in the reaction mechanism depending on whether an electron-withdrawing group or an electron-donating group is attached to the migrating aryl ring. The possibility of the formation of either α,β-ditosyloxy ketone or β,β-ditosyloxy ketone exists. The aryl migration barrier plays a significant role in determining the reaction outcome. The mechanism for formation of α,β-ditosyloxy ketone is found to be the same for chalcones irrespective of whether an electron-donating or electron-withdrawing substituent is present (Scheme 4). But the detailed mechanism for the formation of β,β-ditosyloxy ketone is different and depends on the nature of substituent (Scheme 2 and Scheme 3). The current study shows that for electron-donating groups (-OCH_3_, -SCH_3_), the free energy barrier for the formation of β,β-ditosyloxy ketone is less compared to the formation of α,β-ditosyloxy ketone. However, in case of electron-withdrawing substituents (-Cl, -NO_2_ groups), the free energy barrier is nearly the same for both α,β-ditosyloxy ketone and β,β-ditosyloxy ketone. Therefore, for chalcones with electron-withdrawing substituent on the aryl ring, there are equal chances of getting α,β-ditosyloxy ketone and β,β-ditosyloxy ketone as products. At this point, it is to be noted that for chalcones with X = electron-withdrawing group on the phenyl ring, the interaction of [PhIOH]^+^ with chalcone to form **Int1** is weak as evident from comparatively longer C–I bonds in **Int1** (see Table 1). Therefore, for X = -NO_2_ which is a strong electron-withdrawing group, the reaction between chalcone and HTIB leading to the formation of ditosyloxy ketone may not occur at all under the experimental conditions.

## Supporting Information

File 1 Free energy of intermediates and structures corresponding to various intermediates shown in free energy profiles.

File 2 Coordinates of all structures reported for the first time.

## Data Availability

Data generated and analyzed during this study is available from the corresponding author upon reasonable request.
