# Supplementary material for: Mechanistic insights into hydroxy(tosyloxy)iodobenzene-mediated ditosyloxylation of chalcones: a DFT study
Source: Beilstein J Org Chem. 2025 Dec 16;21:2703–15. doi: 10.3762/bjoc.21.208 (PMC12719883; doi:10.3762/bjoc.21.208)
Supplement: File 1 — Free energy of intermediates and structures corresponding to various intermediates shown in free energy profiles. [file Beilstein_J_Org_Chem-21-2703-s001.pdf]

BEILSTEIN JOURNAL OF ORGANIC CHEMISTRY

Supporting Information

for

Mechanistic insights into hydroxy(tosyloxy)iodobenzene-mediated ditosyloxylation of chalcones: a DFT study

Jai Parkash, Sangeeta Saini, Vaishali Saini, Omkar Bains and Raj Kamal

Beilstein J. Org. Chem. **2025,** *21,* 2703–2715. doi:10.3762/bjoc.21.208

Free energy of intermediates and structures corresponding to various intermediates shown in free energy profiles

License and Terms: This is a supporting information file under the terms of the Creative Commons Attribution License (https://creativecommons.org/licenses/by/4.0). Please note that the reuse, redistribution and reproduction in particular requires that the author(s) and source are credited and that individual graphics may be subject to special legal provisions.
The license is subject to the *Beilstein Journal of Organic Chemistry* terms and conditions: (https://www.beilstein-journals.org/bjoc/terms)

Table S1: Free energy of intermediates involved in ditosyloxylation reaction of chalcones.

**Reactive Intermediates**	**Total Gibbs free energy (a.u.)**
Int2 + $[PhIOH]^+$ + $[OTs]^-$	-3555.421206
Int3 +$[PhIOH]^+$ + $[OTs]^-$ + OH^-	-3555.372379
Int3 + PhIO + H_2O + OTs^-	-3555.452820

The lowering in Gibbs free energy from **Int2** to **Int3** only happens if the hydoxy group of **Int2** interacts with second molecule of dissociated HTIB to form PhIO and H_2O.

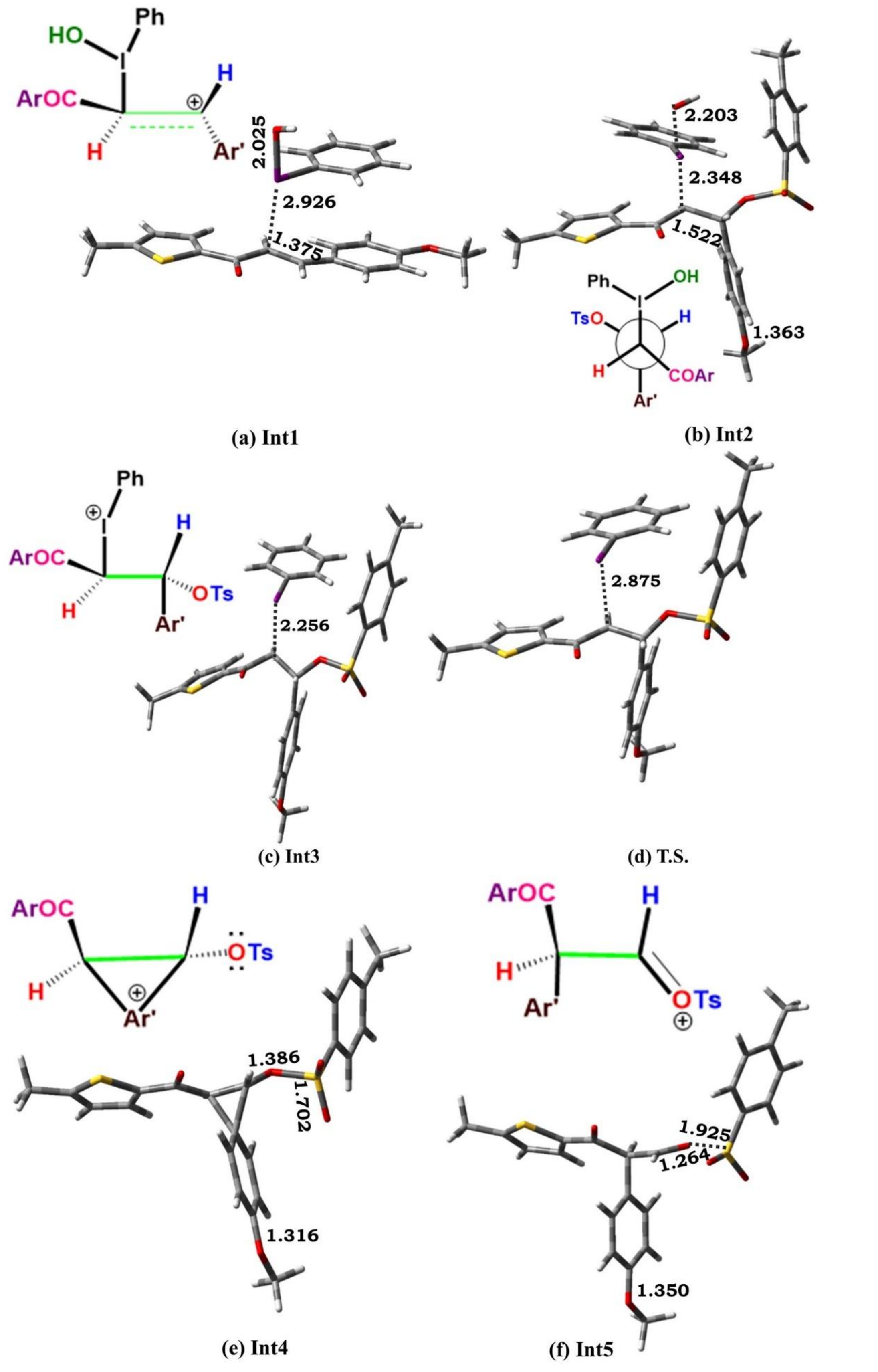

Figure S1: Structures corresponding to various intermediates listed in Table 1 for the reaction of a chalcone with X = -OCH_3.

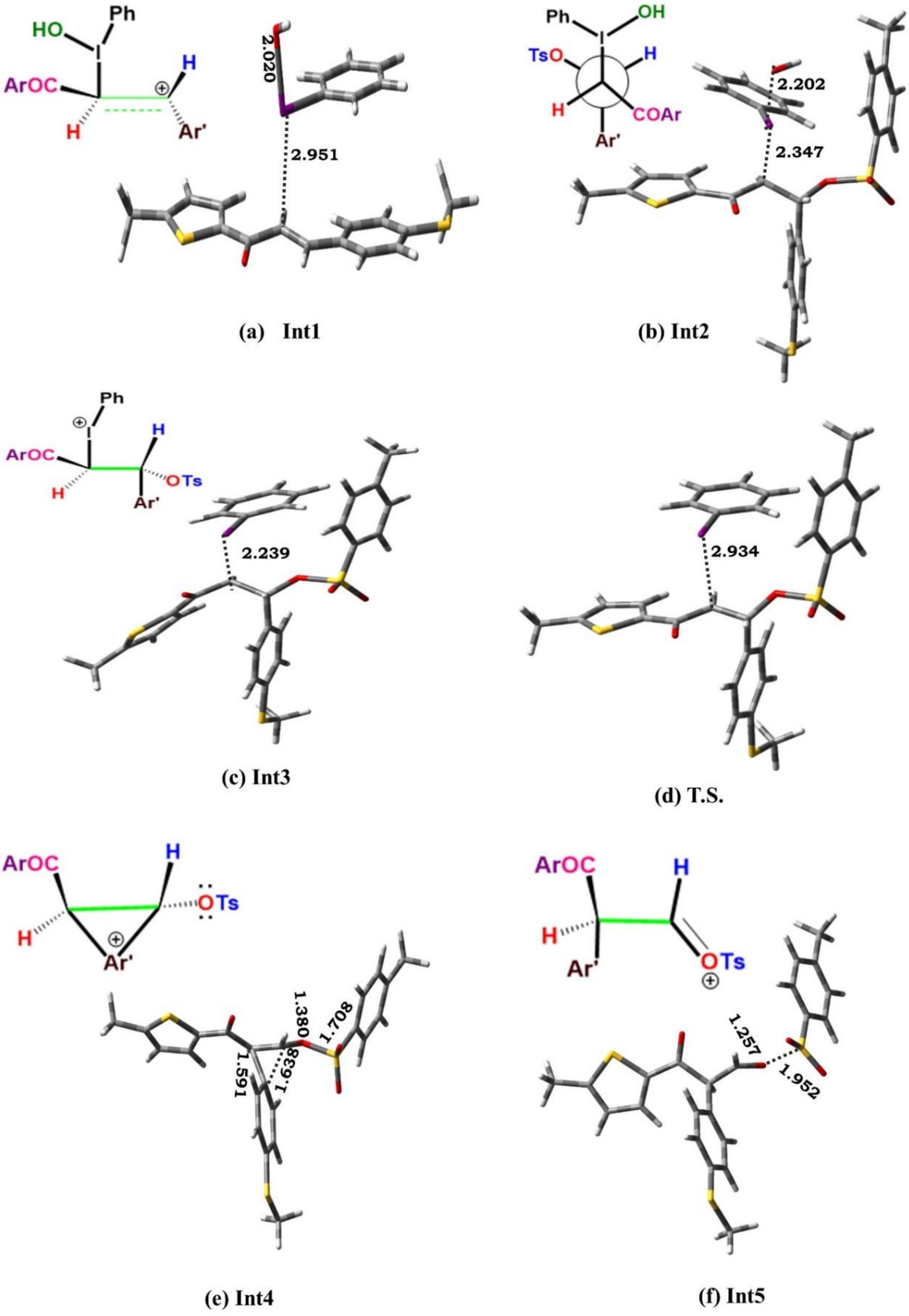

Figure S2: Structures corresponding to various intermediates shown in free energy profile presented in **Figure 1** for X = -SCH_3 leading to formation of β,β-ditosyloxy ketone.

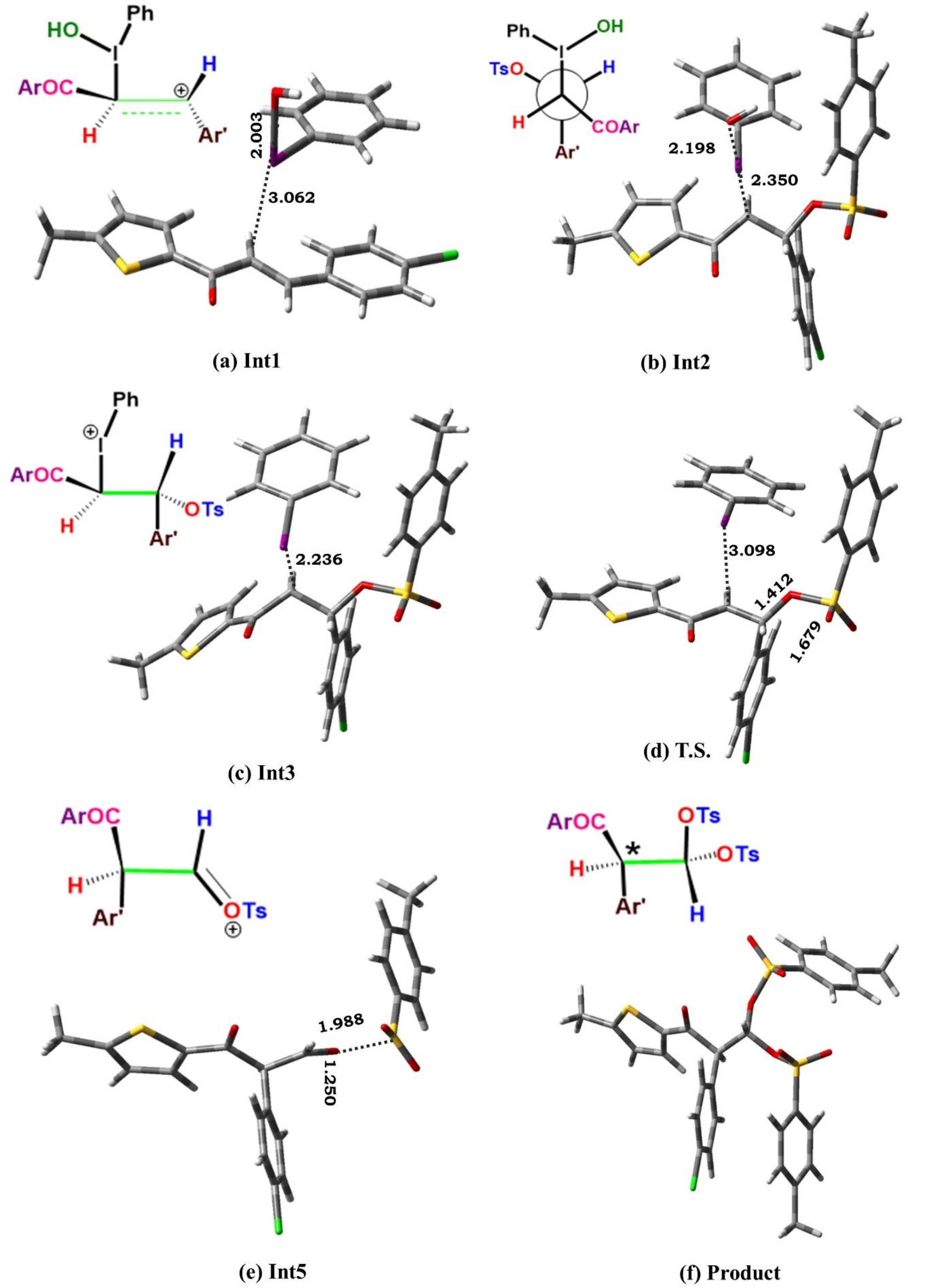

Figure S3: Structures corresponding to various intermediates shown in free energy profile presented in **Figure 2** for X = -Cl leading to formation of β,β-ditosyloxy ketone.

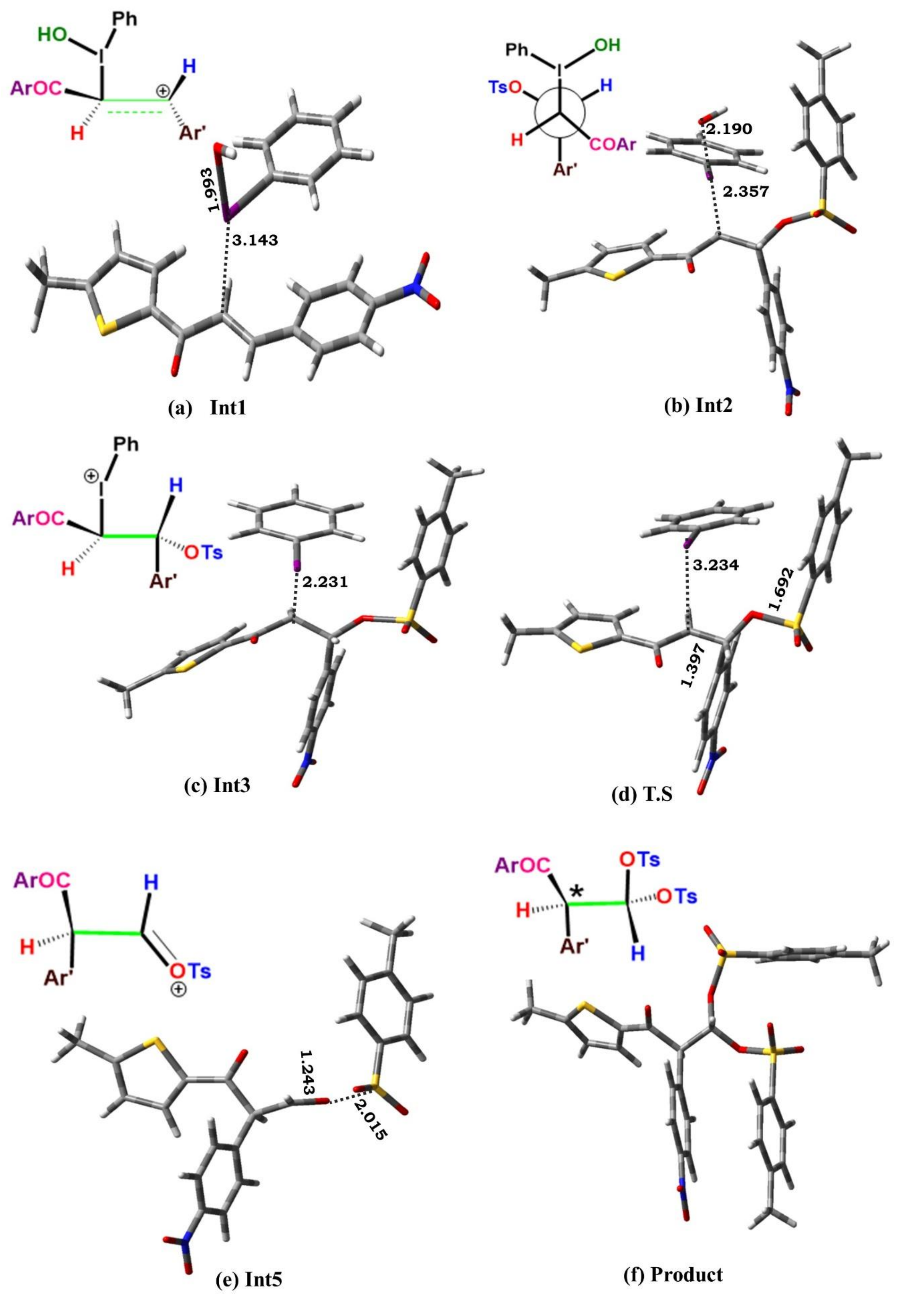

Figure S4: Structures corresponding to various intermediates shown in free energy profile presented in **Figure 3** for X = -NO_2 leading to formation of β,β-ditosyloxy ketone.

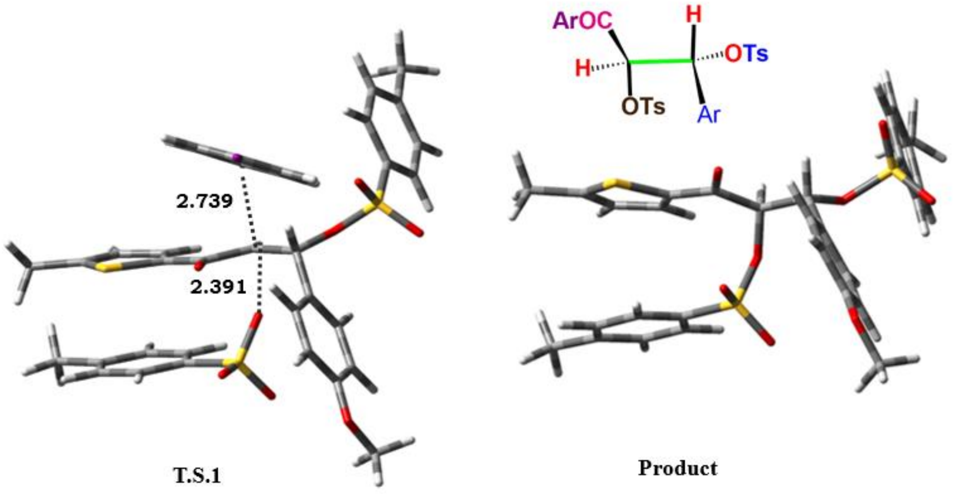

Figure S5: Optimized structure of transition state and product corresponding to free energy profile presented in **Figure 4** for X = -OCH_3. Product is α,β-ditosyloxy ketone. Important bond lengths are reported in Å.

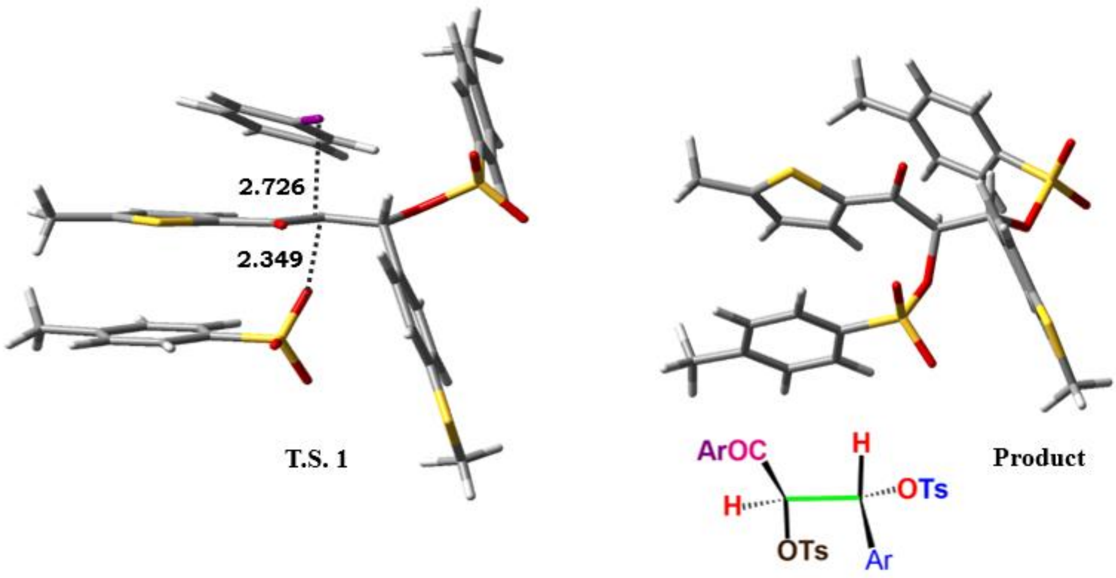

Figure S6: Optimized structure of transition state and product corresponding to free energy profile presented in **Figure 5** for X = -SCH_3. Product is α,β-ditosyloxy ketone. Important bond lengths are reported in Å.

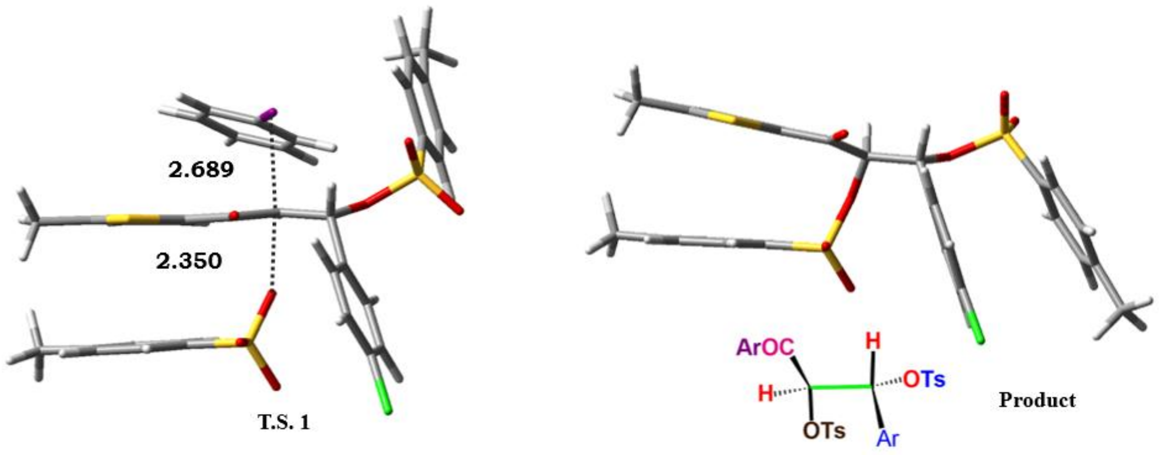

Figure S7: Optimized structure of transition state and product corresponding to free energy profile presented in **Figure 6** for X = -Cl. Product is α,β-ditosyloxy ketone. Important bond lengths are reported in Å.

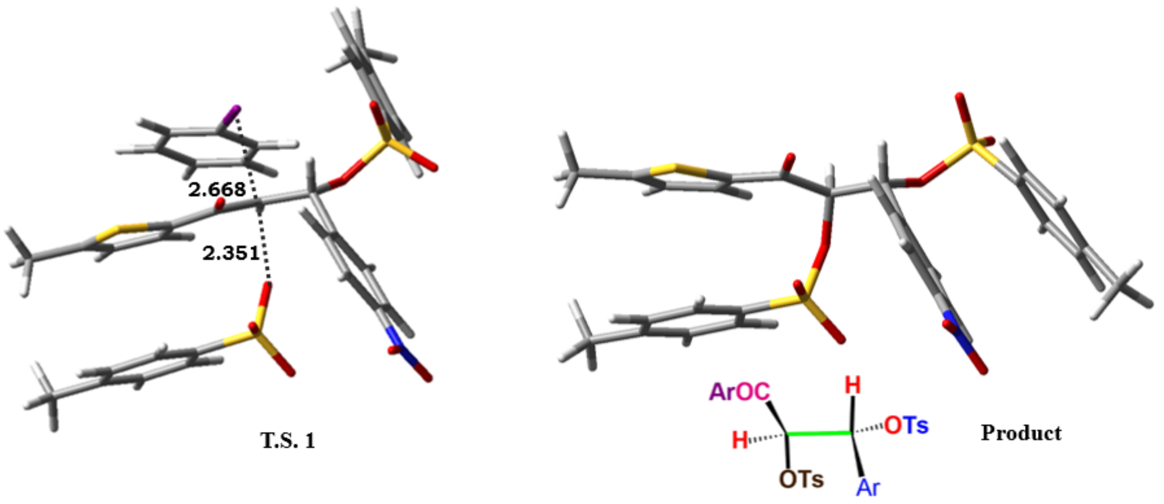

Figure S8: Optimized structure of transition state and product corresponding to free energy profile presented in **Figure 7** for X = -NO_2. Product is α,β-ditosyloxy ketone. Important bond lengths are reported in Å.
