# Supplementary material for: Mechanistic insights into hydroxy(tosyloxy)iodobenzene-mediated ditosyloxylation of chalcones: a DFT study
Source: Beilstein J Org Chem. 2025 Dec 16;21:2703–15. doi: 10.3762/bjoc.21.208 (PMC12719883; doi:10.3762/bjoc.21.208)
Supplement: File 2 — Coordinates of all structures reported for the first time. [file Beilstein_J_Org_Chem-21-2703-s002.pdf]

BEILSTEIN JOURNAL OF ORGANIC CHEMISTRY

Supporting Information

for

Mechanistic insights into hydroxy(tosyloxy)iodobenzene-mediated ditosyloxylation of chalcones: a DFT study

Jai Parkash, Sangeeta Saini, Vaishali Saini, Omkar Bains and Raj Kamal

Beilstein J. Org. Chem. **2025,** *21,* 2703–2715. doi:10.3762/bjoc.21.208

Coordinates of all structures reported for the first time

License and Terms: This is a supporting information file under the terms of the Creative Commons Attribution License (https://creativecommons.org/licenses/by/4.0). Please note that the reuse, redistribution and reproduction in particular requires that the author(s) and source are credited and that individual graphics may be subject to special legal provisions.
The license is subject to the *Beilstein Journal of Organic Chemistry* terms and conditions: (https://www.beilstein-journals.org/bjoc/terms)

COORDINATES OF ALL THE STRUCTURES REPORTED FOR THE FIRST TIME IN THE RESEARCH PAPER

$-SCH_3$ group (Formation of β, β-ditosyloxy ketone)

Reactant

Zero-point correction= 0.247620 (Hartree/Particle)
Thermal correction to Energy= 0.265439
Thermal correction to Enthalpy= 0.266383
Thermal correction to Gibbs Free Energy= 0.199057
Sum of electronic and zero-point Energies= -1451.471273
Sum of electronic and thermal Energies= -1451.453454
Sum of electronic and thermal Enthalpies= -1451.452510
Sum of electronic and thermal Free Energies= -1451.519836

Standard orientation:

Center Number	Atomic Number	Atomic Type	Coordinates (Angstroms) X	Y	Z
1	6	0	-0.737186	-0.404124	-0.006719
2	6	0	0.358084	-1.198746	-0.014206
3	6	0	-2.094933	-0.980858	-0.008626
4	8	0	-2.298951	-2.207691	-0.016665
5	6	0	-3.229122	-0.045553	-0.000507
6	6	0	-3.257725	1.337376	0.007174
7	16	0	-4.862428	-0.691808	-0.000485
8	6	0	-4.572907	1.872163	0.013166
9	1	0	-2.367644	1.955351	0.008289
10	6	0	-5.557545	0.906976	0.010057
11	1	0	-4.795025	2.934033	0.019536
12	6	0	-7.041474	1.108968	0.014523
13	1	0	-7.505742	0.652409	0.896377
14	1	0	-7.269311	2.178634	0.021868
15	1	0	-7.509504	0.663752	-0.871151
16	1	0	-0.641874	0.675446	0.003777
17	1	0	0.187319	-2.274499	-0.018137
18	6	0	1.755564	-0.781731	-0.013611
19	6	0	2.762270	-1.770985	0.017527
20	6	0	2.167668	0.566408	-0.041658
21	6	0	4.110838	-1.435607	0.026348
22	1	0	2.476532	-2.819489	0.037427
23	6	0	3.515091	0.913283	-0.034280
24	1	0	1.430129	1.362756	-0.072928
25	1	0	4.857931	-2.224538	0.053116
26	1	0	3.784409	1.962540	-0.058068
27	6	0	4.506948	-0.084739	0.002005

28	16	0	6.253642	0.246744	0.017111
29	6	0	6.357022	2.068658	0.002914
30	1	0	7.425382	2.299382	0.020437
31	1	0	5.918535	2.486530	-0.906804
32	1	0	5.885482	2.501322	0.888916

Int1

Zero-point correction= 0.352738 (Hartree/Particle)
Thermal correction to Energy= 0.380030
Thermal correction to Enthalpy= 0.380974
Thermal correction to Gibbs Free Energy= 0.292097
Sum of electronic and zero-point Energies= -1770.006501
Sum of electronic and thermal Energies= -1769.979209
Sum of electronic and thermal Enthalpies= -1769.978265
Sum of electronic and thermal Free Energies= -1770.067141
Standard orientation:

Center Number	Atomic Number	Atomic Type	Coordinates (Angstroms) X	Y	Z
1	6	0	0.984457	-1.204669	-0.396442
2	6	0	-0.073902	-1.373387	-1.255538
3	6	0	2.381836	-1.277864	-0.902341
4	8	0	2.627803	-1.346524	-2.117293
5	6	0	3.457446	-1.239153	0.086585
6	6	0	3.399543	-1.172158	1.469170
7	16	0	5.125345	-1.271075	-0.458961
8	6	0	4.677430	-1.144217	2.081144
9	1	0	2.472908	-1.146597	2.030275
10	6	0	5.719470	-1.190549	1.176293
11	1	0	4.837136	-1.093543	3.152865
12	6	0	7.187278	-1.182581	1.470899
13	1	0	7.691165	-0.343742	0.977099
14	1	0	7.347154	-1.094263	2.548895
15	1	0	7.668579	-2.105030	1.125119
16	1	0	0.827126	-1.182631	0.676389
17	1	0	0.167074	-1.427899	-2.316450
18	6	0	-1.470844	-1.459485	-0.911501
19	6	0	-2.430809	-1.424967	-1.951686
20	6	0	-1.940346	-1.556583	0.418235
21	6	0	-3.788540	-1.452257	-1.678064
22	1	0	-2.094705	-1.362233	-2.983303
23	6	0	-3.297173	-1.596571	0.700650
24	1	0	-1.238003	-1.606889	1.243592
25	6	0	-4.242771	-1.530371	-0.343737
26	1	0	-4.502012	-1.411911	-2.496694
27	1	0	-3.616194	-1.669028	1.733256
28	6	0	-6.182882	-1.656059	1.714230

29	1	0	-5.736029	-0.796363	2.219618
30	1	0	-7.261436	-1.653198	1.892798
31	1	0	-5.761639	-2.589240	2.096377
32	6	0	-3.340021	1.865593	0.389468
33	6	0	-2.142163	1.829710	-0.326524
34	6	0	-0.946743	1.728246	0.394756
35	6	0	-0.907318	1.645866	1.791865
36	6	0	-2.119578	1.682514	2.484587
37	6	0	-3.327718	1.793477	1.786449
38	1	0	-4.280201	1.938980	-0.147910
39	1	0	-2.141537	1.876761	-1.409593
40	1	0	0.034490	1.563975	2.323150
41	1	0	-2.116140	1.624167	3.568790
42	1	0	-4.264956	1.818565	2.334332
43	53	0	0.864293	1.732350	-0.666198
44	8	0	1.098067	3.738827	-0.652039
45	1	0	0.626310	4.096373	-1.429611
46	16	0	-5.991188	-1.534953	-0.095217

Int2

Zero-point correction= 0.485427 (Hartree/Particle)
Thermal correction to Energy= 0.524027
Thermal correction to Enthalpy= 0.524971
Thermal correction to Gibbs Free Energy= 0.407593
Sum of electronic and zero-point Energies= -2664.866876
Sum of electronic and thermal Energies= -2664.828276
Sum of electronic and thermal Enthalpies= -2664.827332
Sum of electronic and thermal Free Energies= -2664.944710

Standard orientation:

Center Number	Atomic Number	Atomic Type	Coordinates (Angstroms) X	Y	Z
1	6	0	0.603764	0.478416	-0.432612
2	6	0	0.734142	-0.981240	-0.847384
3	6	0	1.617702	1.379145	-1.046837
4	8	0	2.083537	1.150608	-2.174634
5	6	0	1.993854	2.585746	-0.300892
6	6	0	1.624025	2.998871	0.965793
7	16	0	3.064833	3.760522	-1.044236
8	6	0	2.192145	4.246390	1.335889
9	1	0	0.962418	2.430824	1.609383
10	6	0	2.999177	4.791996	0.359493
11	1	0	2.018941	4.732011	2.290562
12	6	0	3.756413	6.083646	0.409713
13	1	0	3.434229	6.768367	-0.383624
14	1	0	3.589288	6.572585	1.373806

15	1	0	4.833966	5.922432	0.287952
16	1	0	0.538745	0.570965	0.650062
17	6	0	-2.538335	0.790005	2.984314
18	6	0	-2.196453	0.554525	1.648407
19	6	0	-1.958341	1.649929	0.817549
20	6	0	-2.046589	2.965437	1.276307
21	6	0	-2.387440	3.181652	2.616205
22	6	0	-2.632936	2.099035	3.468178
23	1	0	-2.733623	-0.054688	3.638904
24	1	0	-2.114319	-0.456317	1.269423
25	1	0	-1.858281	3.802386	0.612841
26	1	0	-2.456919	4.199417	2.989602
27	1	0	-2.898604	2.275899	4.506474
28	53	0	-1.452119	1.300306	-1.213810
29	8	0	-3.452814	2.085622	-1.694796
30	1	0	-4.103619	1.416830	-1.423726
31	1	0	0.585867	-1.088494	-1.923913
32	6	0	2.048132	-1.605541	-0.439677
33	6	0	2.943086	-2.072251	-1.410390
34	6	0	2.406589	-1.704234	0.909887
35	6	0	4.170405	-2.621097	-1.042707
36	1	0	2.681414	-2.003787	-2.462602
37	6	0	3.629971	-2.257402	1.291253
38	1	0	1.722600	-1.353412	1.677915
39	6	0	4.528463	-2.719106	0.314233
40	1	0	4.848525	-2.976444	-1.814455
41	1	0	3.868743	-2.320563	2.346438
42	6	0	6.199910	-3.421068	2.500743
43	1	0	5.400946	-4.025459	2.938753
44	1	0	7.163942	-3.869400	2.755870
45	1	0	6.167380	-2.402192	2.895738
46	8	0	-0.374751	-1.654732	-0.142480
47	16	0	-1.131859	-2.940496	-0.821415
48	8	0	-0.648469	-4.160245	-0.165608
49	8	0	-1.038649	-2.823559	-2.283334
50	6	0	-2.783130	-2.572390	-0.269677
51	6	0	-3.208644	-3.045086	0.973211
52	6	0	-3.592703	-1.750104	-1.060712
53	6	0	-4.470600	-2.667426	1.435772
54	1	0	-2.567402	-3.687357	1.567647
55	6	0	-4.848186	-1.387897	-0.579936
56	1	0	-3.245657	-1.401136	-2.026400
57	6	0	-5.302643	-1.829775	0.676187
58	1	0	-4.810913	-3.028215	2.402338
59	1	0	-5.486249	-0.751149	-1.187473
60	6	0	-6.650229	-1.393214	1.191051
61	1	0	-7.426220	-1.531860	0.430230
62	1	0	-6.938436	-1.950173	2.087039
63	1	0	-6.633750	-0.325971	1.445611

64	16	0	6.121973	-3.434063	0.678414

Int3

Zero-point correction= 0.473769 (Hartree/Particle)
Thermal correction to Energy= 0.509978
Thermal correction to Enthalpy= 0.510922
Thermal correction to Gibbs Free Energy= 0.398915
Sum of electronic and zero-point Energies= -2588.904474
Sum of electronic and thermal Energies= -2588.868265
Sum of electronic and thermal Enthalpies= -2588.867321
Sum of electronic and thermal Free Energies= -2588.979328

Standard orientation:

Center	Atomic	Atomic	Coordinates (Angstroms)		
Number	Number	Type	X	Y	Z
1	6	0	-0.423744	-0.824208	-0.470032
2	6	0	-0.244704	0.635378	-0.889447
3	6	0	-1.591438	-1.483048	-1.213329
4	8	0	-1.491610	-1.663196	-2.430341
5	6	0	-2.771941	-1.802902	-0.445657
6	6	0	-3.011739	-1.669389	0.917044
7	16	0	-4.205606	-2.391199	-1.272646
8	6	0	-4.322992	-2.039607	1.286605
9	1	0	-2.274347	-1.306155	1.622946
10	6	0	-5.099528	-2.450727	0.217468
11	1	0	-4.701078	-2.003928	2.302208
12	6	0	-6.525231	-2.902713	0.247421
13	1	0	-6.627233	-3.926807	-0.129949
14	1	0	-6.900760	-2.872213	1.273571
15	1	0	-7.158786	-2.258023	-0.372755
16	1	0	-0.452890	-0.934778	0.613444
17	6	0	3.695105	-1.243088	2.646289
18	6	0	3.208436	-1.207605	1.335922
19	6	0	2.166727	-2.073720	1.001513
20	6	0	1.598885	-2.970229	1.910644
21	6	0	2.099227	-2.981555	3.216642
22	6	0	3.142548	-2.123386	3.581958
23	1	0	4.504093	-0.576072	2.927704
24	1	0	3.624414	-0.523606	0.608411
25	1	0	0.795029	-3.637726	1.620163
26	1	0	1.672814	-3.667460	3.942574
27	1	0	3.525855	-2.142479	4.597774
28	53	0	1.398202	-2.024497	-0.974910
29	1	0	-0.089045	0.698569	-1.969644
30	6	0	-1.486762	1.392860	-0.477740
31	6	0	-2.469189	1.692958	-1.431714

32	6	0	-1.722128	1.702693	0.866478
33	6	0	-3.669211	2.281655	-1.046361
34	1	0	-2.298626	1.457000	-2.478653
35	6	0	-2.917318	2.302475	1.261006
36	1	0	-0.964394	1.481171	1.612520
37	6	0	-3.910797	2.587611	0.307379
38	1	0	-4.419144	2.504334	-1.800602
39	1	0	-3.063838	2.536356	2.308737
40	6	0	-5.419005	3.566780	2.500548
41	1	0	-4.614910	4.251840	2.782000
42	1	0	-6.378803	4.016462	2.768593
43	1	0	-5.313277	2.614833	3.027538
44	8	0	0.945738	1.087834	-0.194528
45	16	0	1.692129	2.468610	-0.738186
46	8	0	1.324383	3.558660	0.166425
47	8	0	1.417728	2.582903	-2.175389
48	6	0	3.380228	1.992474	-0.461179
49	6	0	3.999341	2.338190	0.742449
50	6	0	4.042853	1.253853	-1.447124
51	6	0	5.314893	1.926945	0.957141
52	1	0	3.466459	2.911439	1.493447
53	6	0	5.356218	0.853997	-1.209549
54	1	0	3.544848	0.998116	-2.376268
55	6	0	6.012266	1.182743	-0.009175
56	1	0	5.806815	2.188317	1.889892
57	1	0	5.881657	0.280280	-1.968026
58	6	0	7.443082	0.769661	0.220038
59	1	0	8.125714	1.526576	-0.187268
60	1	0	7.664532	0.666524	1.286548
61	1	0	7.669357	-0.177397	-0.279541
62	16	0	-5.482994	3.323525	0.694214

T.S. 1

Zero-point correction= 0.472484 (Hartree/Particle)
Thermal correction to Energy= 0.508504
Thermal correction to Enthalpy= 0.509448
Thermal correction to Gibbs Free Energy= 0.398250
Sum of electronic and zero-point Energies= -2588.894990
Sum of electronic and thermal Energies= -2588.858970
Sum of electronic and thermal Enthalpies= -2588.858026
Sum of electronic and thermal Free Energies= -2588.969225

Standard orientation:

Center Number	Atomic Number	Atomic Type	Coordinates (Angstroms) X	Y	Z
1	6	0	-0.892952	-0.245073	-0.513007

2	6	0	-0.332688	0.978539	-1.084711
3	6	0	-1.904808	-1.044882	-1.278151
4	8	0	-2.018729	-0.876724	-2.498405
5	6	0	-2.697510	-1.988404	-0.517292
6	6	0	-2.715780	-2.234197	0.850849
7	16	0	-3.840801	-3.024560	-1.350282
8	6	0	-3.633746	-3.241709	1.218659
9	1	0	-2.096003	-1.707845	1.566707
10	6	0	-4.323823	-3.775363	0.144088
11	1	0	-3.794152	-3.575060	2.237990
12	6	0	-5.363879	-4.849408	0.171165
13	1	0	-5.070251	-5.704962	-0.448121
14	1	0	-5.509700	-5.197459	1.197177
15	1	0	-6.323440	-4.481218	-0.210334
16	1	0	-0.737311	-0.412816	0.546474
17	6	0	2.870398	-0.875229	2.972322
18	6	0	2.580063	-1.006783	1.610727
19	6	0	1.729262	-2.038567	1.201318
20	6	0	1.169755	-2.937759	2.115554
21	6	0	1.472040	-2.789751	3.474037
22	6	0	2.317922	-1.761515	3.903420
23	1	0	3.530303	-0.076577	3.298894
24	1	0	3.003515	-0.318361	0.890358
25	1	0	0.515763	-3.736696	1.782404
26	1	0	1.044681	-3.483555	4.192490
27	1	0	2.548530	-1.653488	4.959304
28	53	0	1.251188	-2.220251	-0.851125
29	1	0	-0.264645	0.990901	-2.172084
30	6	0	-1.535440	1.754448	-0.556079
31	6	0	-2.633999	2.040016	-1.405598
32	6	0	-1.553549	2.211540	0.783628
33	6	0	-3.681729	2.806295	-0.943774
34	1	0	-2.637262	1.667426	-2.424263
35	6	0	-2.603337	2.984042	1.247712
36	1	0	-0.717221	1.986079	1.437292
37	6	0	-3.681450	3.298920	0.389214
38	1	0	-4.505785	3.043552	-1.610376
39	1	0	-2.579998	3.343019	2.269098
40	6	0	-4.697707	4.787595	2.567884
41	1	0	-3.763943	5.352952	2.621892
42	1	0	-5.528406	5.437328	2.854497
43	1	0	-4.669627	3.924530	3.237421
44	8	0	0.900090	1.295784	-0.446243
45	16	0	1.797247	2.565664	-1.053607
46	8	0	1.507709	3.747426	-0.239745
47	8	0	1.570207	2.603911	-2.502063
48	6	0	3.398715	1.915313	-0.660772
49	6	0	4.029226	2.323945	0.515874
50	6	0	3.960547	0.952001	-1.507876

51	6	0	5.253823	1.743246	0.850259
52	1	0	3.575133	3.071967	1.156647
53	6	0	5.180471	0.386665	-1.151048
54	1	0	3.453221	0.647608	-2.417105
55	6	0	5.842814	0.766966	0.031920
56	1	0	5.755657	2.053093	1.762419
57	1	0	5.627853	-0.363676	-1.797159
58	6	0	7.153158	0.123121	0.403730
59	1	0	7.017160	-0.950923	0.579882
60	1	0	7.882169	0.226334	-0.408122
61	1	0	7.577937	0.566687	1.308316
62	16	0	-5.053704	4.281221	0.852968

Int4

Zero-point correction= 0.381592 (Hartree/Particle)
Thermal correction to Energy= 0.410440
Thermal correction to Enthalpy= 0.411384
Thermal correction to Gibbs Free Energy= 0.317066
Sum of electronic and zero-point Energies= -2345.946836
Sum of electronic and thermal Energies= -2345.917989
Sum of electronic and thermal Enthalpies= -2345.917045
Sum of electronic and thermal Free Energies= -2346.011363

Standard orientation:

Center Number	Atomic Number	Atomic Type	Coordinates (Angstroms) X	Y	Z
1	6	0	-0.938533	-0.827660	-0.011283
2	6	0	0.235819	-0.389944	-0.730313
3	6	0	-2.043724	-1.565493	-0.744501
4	8	0	-1.958902	-1.741820	-1.961985
5	6	0	-3.171747	-2.011278	0.048124
6	6	0	-3.438110	-1.827653	1.397968
7	16	0	-4.475330	-2.897654	-0.726177
8	6	0	-4.669717	-2.392596	1.801124
9	1	0	-2.776231	-1.299838	2.075359
10	6	0	-5.353558	-3.012072	0.771647
11	1	0	-5.052853	-2.353476	2.814683
12	6	0	-6.673783	-3.712204	0.843725
13	1	0	-6.586190	-4.760627	0.536618
14	1	0	-7.051903	-3.682621	1.868956
15	1	0	-7.412579	-3.237337	0.187881
16	1	0	-0.791830	-1.075585	1.034381
17	1	0	0.279513	-0.474325	-1.812393
18	6	0	-0.846298	0.742786	-0.249848
19	6	0	-1.597492	1.362303	-1.313993
20	6	0	-0.494720	1.549579	0.888992

21	6	0	-1.953266	2.678784	-1.240607
22	1	0	-1.867373	0.774892	-2.185344
23	6	0	-0.862458	2.866306	0.963691
24	1	0	0.081316	1.103714	1.692326
25	6	0	-1.597106	3.460832	-0.100206
26	1	0	-2.505655	3.133353	-2.056934
27	1	0	-0.575160	3.446126	1.831336
28	6	0	-1.478259	5.831503	1.432980
29	1	0	-0.387599	5.787906	1.472366
30	1	0	-1.798092	6.875945	1.411373
31	1	0	-1.924584	5.334139	2.296766
32	8	0	1.437129	-0.453675	-0.052725
33	16	0	2.678904	0.587883	-0.593494
34	8	0	2.578735	1.791446	0.231502
35	8	0	2.549757	0.676320	-2.048840
36	6	0	4.065741	-0.398456	-0.116606
37	6	0	4.632961	-0.206013	1.147124
38	6	0	4.546922	-1.360485	-1.010547
39	6	0	5.713708	-1.003941	1.514531
40	1	0	4.243118	0.548041	1.822083
41	6	0	5.629326	-2.143747	-0.618225
42	1	0	4.090401	-1.491619	-1.985833
43	6	0	6.228491	-1.979489	0.643017
44	1	0	6.166172	-0.866082	2.492380
45	1	0	6.016606	-2.893276	-1.302195
46	6	0	7.419968	-2.809632	1.042641
47	1	0	7.437205	-3.766532	0.513092
48	1	0	8.348880	-2.278653	0.797744
49	1	0	7.427502	-3.002453	2.119729
50	16	0	-2.099788	5.115493	-0.125616

Int5

Zero-point correction= 0.380998 (Hartree/Particle)
Thermal correction to Energy= 0.410324
Thermal correction to Enthalpy= 0.411268
Thermal correction to Gibbs Free Energy= 0.316047
Sum of electronic and zero-point Energies= -2345.951903
Sum of electronic and thermal Energies= -2345.922578
Sum of electronic and thermal Enthalpies= -2345.921634
Sum of electronic and thermal Free Energies= -2346.016855

Standard orientation:

Center Number	Atomic Number	Atomic Type	Coordinates (Angstroms) X	Y	Z
1	6	0	0.513506	0.450004	0.414105
2	6	0	-0.659802	-0.193759	-0.230839

3	6	0	0.756922	1.819844	-0.243884
4	8	0	-0.086607	2.242007	-1.041740
5	6	0	1.944835	2.552797	0.132370
6	6	0	2.914798	2.225025	1.067194
7	16	0	2.303501	4.097509	-0.622286
8	6	0	3.930953	3.202069	1.175608
9	1	0	2.895074	1.311475	1.649684
10	6	0	3.752120	4.277357	0.325455
11	1	0	4.776320	3.127669	1.850495
12	6	0	4.636181	5.474519	0.168692
13	1	0	4.094063	6.402756	0.382365
14	1	0	5.481067	5.401193	0.858372
15	1	0	5.028438	5.548946	-0.852062
16	1	0	0.335059	0.583681	1.484817
17	1	0	-0.693703	-0.277820	-1.321912
18	6	0	1.568909	-0.633014	0.183189
19	6	0	2.213959	-0.763272	-1.060231
20	6	0	1.873195	-1.539600	1.208712
21	6	0	3.169373	-1.751069	-1.255359
22	1	0	1.983780	-0.080134	-1.872765
23	6	0	2.832787	-2.531282	1.020412
24	1	0	1.366228	-1.460834	2.166506
25	6	0	3.495909	-2.645953	-0.215402
26	1	0	3.666621	-1.827249	-2.218053
27	1	0	3.055726	-3.205326	1.838611
28	6	0	4.888585	-4.805608	0.983216
29	1	0	3.952147	-5.309093	1.236217
30	1	0	5.655832	-5.559613	0.789457
31	1	0	5.217816	-4.167081	1.806761
32	8	0	-1.561090	-0.721446	0.469092
33	16	0	-2.926288	-1.803684	-0.412848
34	8	0	-2.869052	-2.967477	0.451549
35	8	0	-2.478324	-1.797197	-1.797315
36	6	0	-4.295615	-0.768003	-0.115525
37	6	0	-4.977000	-0.896552	1.103342
38	6	0	-4.643467	0.184514	-1.087887
39	6	0	-6.053910	-0.048872	1.336136
40	1	0	-4.679124	-1.635530	1.838521
41	6	0	-5.723641	1.013153	-0.821784
42	1	0	-4.092557	0.265303	-2.018274
43	6	0	-6.444015	0.912188	0.385730
44	1	0	-6.602079	-0.135259	2.268976
45	1	0	-6.019209	1.751704	-1.560781
46	6	0	-7.616940	1.817817	0.634574
47	1	0	-8.011291	1.701496	1.646860
48	1	0	-7.335040	2.865783	0.483393
49	1	0	-8.422024	1.596561	-0.076798
50	16	0	4.737887	-3.860048	-0.569868

Product

Zero-point correction= 0.515946 (Hartree/Particle)
Thermal correction to Energy= 0.555665
Thermal correction to Enthalpy= 0.556610
Thermal correction to Gibbs Free Energy= 0.437717
Sum of electronic and zero-point Energies= -3240.877183
Sum of electronic and thermal Energies= -3240.837464
Sum of electronic and thermal Enthalpies= -3240.836520
Sum of electronic and thermal Free Energies= -3240.955412

Standard orientation:

Center Number	Atomic Number	Atomic Type	Coordinates (Angstroms) X	Y	Z
1	6	0	-0.025098	1.242705	0.010731
2	1	0	-0.293983	1.182388	1.066942
3	6	0	-0.786665	0.118752	-0.699197
4	1	0	-0.623470	0.111948	-1.776228
5	6	0	1.481067	1.089266	-0.126165
6	6	0	2.114540	1.189282	-1.374808
7	6	0	2.268368	0.839276	1.001911
8	6	0	3.493025	1.037944	-1.488699
9	1	0	1.525940	1.384407	-2.265571
10	6	0	3.653750	0.690707	0.900279
11	1	0	1.798677	0.748620	1.977570
12	6	0	4.279786	0.784031	-0.350557
13	1	0	3.955586	1.105487	-2.469854
14	1	0	4.224925	0.490184	1.798925
15	8	0	-0.399136	-1.137672	-0.124122
16	16	0	0.246244	-2.353466	-1.060006
17	8	0	0.545070	-1.825798	-2.392636
18	8	0	-0.660819	-3.493553	-0.902456
19	6	0	1.751901	-2.682272	-0.172136
20	6	0	2.973429	-2.396926	-0.778273
21	6	0	1.674893	-3.249543	1.105856
22	6	0	4.148278	-2.693742	-0.084167
23	1	0	3.009732	-1.947795	-1.763581
24	6	0	2.858463	-3.525702	1.781929
25	1	0	0.714369	-3.472845	1.558610
26	6	0	4.111289	-3.257455	1.197827
27	1	0	5.104189	-2.469613	-0.547336
28	1	0	2.813607	-3.963652	2.775479
29	6	0	5.379313	-3.563615	1.953551
30	1	0	5.384553	-4.601753	2.304354
31	1	0	6.265213	-3.405561	1.332923
32	1	0	5.464901	-2.924030	2.840528
33	8	0	-2.159144	0.337405	-0.381789

34	16	0	-3.358334	-0.274332	-1.368751
35	8	0	-2.705397	-1.047586	-2.428924
36	8	0	-4.210867	0.868667	-1.694597
37	6	0	-4.211818	-1.350200	-0.239692
38	6	0	-3.752833	-2.659970	-0.077992
39	6	0	-5.307265	-0.857138	0.474062
40	6	0	-4.417935	-3.489960	0.822290
41	1	0	-2.894393	-3.019843	-0.633462
42	6	0	-5.955676	-1.707340	1.368810
43	1	0	-5.646048	0.163209	0.331705
44	6	0	-5.526101	-3.031960	1.555419
45	1	0	-4.070250	-4.510682	0.955822
46	1	0	-6.809662	-1.336087	1.928467
47	6	0	-6.258122	-3.952509	2.497804
48	1	0	-7.052458	-4.489838	1.963788
49	1	0	-5.585638	-4.702248	2.925678
50	1	0	-6.728948	-3.396861	3.314426
51	6	0	-0.473004	2.589477	-0.581712
52	8	0	-0.652962	2.692368	-1.797183
53	6	0	-0.623208	3.722329	0.320895
54	6	0	-0.496169	3.785889	1.697812
55	16	0	-1.028214	5.301303	-0.335151
56	6	0	-0.719829	5.084215	2.219556
57	1	0	-0.251964	2.930082	2.315912
58	6	0	-1.018772	6.020723	1.250668
59	1	0	-0.666569	5.331737	3.274195
60	6	0	-1.307117	7.477032	1.444400
61	1	0	-0.570871	8.101986	0.925622
62	1	0	-2.297240	7.744352	1.057883
63	1	0	-1.275666	7.721109	2.509835
64	6	0	6.653096	0.091686	1.034963
65	1	0	7.720427	-0.103687	0.901543
66	1	0	6.528681	0.895098	1.765574
67	1	0	6.165296	-0.820649	1.385566
68	16	0	6.032029	0.578620	-0.609457

Chloride Group (Formation of β, β-ditosyloxy ketone)

Reactant

Zero-point correction= 0.209237 (Hartree/Particle)
Thermal correction to Energy= 0.224999
Thermal correction to Enthalpy= 0.225943
Thermal correction to Gibbs Free Energy= 0.163570
Sum of electronic and zero-point Energies= -1473.592989
Sum of electronic and thermal Energies= -1473.577227
Sum of electronic and thermal Enthalpies= -1473.576283
Sum of electronic and thermal Free Energies= -1473.638656

Standard orientation:

Center Number	Atomic Number	Atomic Type	Coordinates (Angstroms) X	Y	Z
1	6	0	-0.334076	0.280862	-0.000005
2	6	0	0.798231	1.016987	-0.000017
3	6	0	-1.662176	0.932754	-0.000010
4	8	0	-1.792657	2.168237	-0.000025
5	6	0	-2.845592	0.063876	0.000005
6	6	0	-2.952970	-1.315973	0.000021
7	16	0	-4.439532	0.802651	0.000004
8	6	0	-4.296028	-1.774135	0.000033
9	1	0	-2.100314	-1.984960	0.000024
10	6	0	-5.223851	-0.753735	0.000026
11	1	0	-4.578971	-2.821368	0.000046
12	6	0	-6.716667	-0.870840	0.000036
13	1	0	-7.155848	-0.393735	-0.883779
14	1	0	-7.005280	-1.925624	0.000053
15	1	0	-7.155839	-0.393709	0.883843
16	1	0	-0.298063	-0.802197	0.000009
17	1	0	0.685964	2.100163	-0.000030
18	6	0	2.176321	0.525865	-0.000014
19	6	0	3.224663	1.467313	-0.000028
20	6	0	2.513653	-0.844276	0.000003
21	6	0	4.561515	1.070119	-0.000026
22	1	0	2.988889	2.527904	-0.000041
23	6	0	3.842255	-1.257423	0.000005
24	1	0	1.736930	-1.602081	0.000015
25	6	0	4.854998	-0.293043	-0.000010
26	1	0	5.357694	1.806898	-0.000038
27	1	0	4.090762	-2.313309	0.000018
28	17	0	6.539188	-0.816259	-0.000007

Int1

Zero-point correction= 0.314605 (Hartree/Particle)
Thermal correction to Energy= 0.339799
Thermal correction to Enthalpy= 0.340743
Thermal correction to Gibbs Free Energy= 0.255717
Sum of electronic and zero-point Energies= -1792.125088
Sum of electronic and thermal Energies= -1792.099894
Sum of electronic and thermal Enthalpies= -1792.098950
Sum of electronic and thermal Free Energies= -1792.183976

Standard orientation:

Center Number	Atomic Number	Atomic Type	Coordinates (Angstroms) X	Y	Z

1	6	0	0.888378	-1.193921	-0.386862
2	6	0	-0.176149	-1.437591	-1.204674
3	6	0	2.274966	-1.155805	-0.928697
4	8	0	2.493501	-1.169374	-2.150014
5	6	0	3.366876	-1.098733	0.039658
6	6	0	3.334004	-1.094746	1.425670
7	16	0	5.023945	-1.040383	-0.536593
8	6	0	4.621159	-1.042073	2.014572
9	1	0	2.419324	-1.131475	2.005647
10	6	0	5.646265	-1.008110	1.089631
11	1	0	4.800020	-1.031833	3.084370
12	6	0	7.117923	-0.957233	1.358722
13	1	0	7.580577	-0.078198	0.895603
14	1	0	7.294559	-0.912746	2.436890
15	1	0	7.626059	-1.844150	0.962123
16	1	0	0.758398	-1.142831	0.688142
17	1	0	0.023805	-1.498465	-2.273499
18	6	0	-1.560731	-1.617845	-0.795359
19	6	0	-2.562210	-1.626279	-1.788103
20	6	0	-1.949178	-1.763194	0.553934
21	6	0	-3.909806	-1.740367	-1.452907
22	1	0	-2.280672	-1.529853	-2.833029
23	6	0	-3.287965	-1.889317	0.902024
24	1	0	-1.203661	-1.786109	1.341193
25	6	0	-4.257437	-1.863095	-0.106265
26	1	0	-4.674476	-1.734793	-2.222323
27	1	0	-3.579917	-1.997116	1.940598
28	6	0	-3.607936	1.563269	0.970693
29	6	0	-2.556770	1.647275	0.056347
30	6	0	-1.248391	1.613367	0.553312
31	6	0	-0.955637	1.479130	1.915700
32	6	0	-2.025484	1.390097	2.808996
33	6	0	-3.343003	1.435198	2.338598
34	1	0	-4.631374	1.585157	0.609851
35	1	0	-2.752567	1.732856	-1.006636
36	1	0	0.069052	1.452315	2.270063
37	1	0	-1.825728	1.288357	3.871345
38	1	0	-4.167882	1.363323	3.041200
39	53	0	0.338025	1.789681	-0.805116
40	8	0	0.361769	3.792409	-0.842965
41	1	0	-0.245529	4.083807	-1.551899
42	17	0	-5.953168	-1.991156	0.338287

Int2

Zero-point correction= 0.447138 (Hartree/Particle)
Thermal correction to Energy= 0.483609
Thermal correction to Enthalpy= 0.484554

Thermal correction to Gibbs Free Energy= 0.371942
Sum of electronic and zero-point Energies= -2686.990191
Sum of electronic and thermal Energies= -2686.953720
Sum of electronic and thermal Enthalpies= -2686.952776
Sum of electronic and thermal Free Energies= -2687.065387

Standard orientation:

Center Number	Atomic Number	Atomic Type	Coordinates (Angstroms) X	Y	Z
1	6	0	-0.844342	-0.217829	-0.317816
2	6	0	-0.643177	1.254693	-0.650527
3	6	0	-2.021483	-0.825986	-0.997169
4	8	0	-2.397461	-0.422305	-2.109923
5	6	0	-2.683945	-1.959710	-0.342995
6	6	0	-2.476784	-2.519694	0.904441
7	16	0	-3.959559	-2.811743	-1.196387
8	6	0	-3.330821	-3.621965	1.171892
9	1	0	-1.738085	-2.157550	1.609337
10	6	0	-4.194996	-3.910055	0.136183
11	1	0	-3.317652	-4.188015	2.097403
12	6	0	-5.230476	-4.991107	0.077400
13	1	0	-5.046738	-5.678330	-0.756744
14	1	0	-5.216497	-5.567579	1.006786
15	1	0	-6.236560	-4.575632	-0.053940
16	1	0	-0.823436	-0.382327	0.757593
17	6	0	2.197022	-1.372410	3.074101
18	6	0	1.937817	-1.004211	1.749340
19	6	0	1.399235	-1.959453	0.885700
20	6	0	1.111484	-3.260873	1.301830
21	6	0	1.376300	-3.610061	2.630674
22	6	0	1.916545	-2.669952	3.514854
23	1	0	2.620376	-0.639850	3.755536
24	1	0	2.148243	0.002133	1.408859
25	1	0	0.694176	-3.987327	0.613069
26	1	0	1.155808	-4.618288	2.969765
27	1	0	2.119593	-2.948790	4.544861
28	53	0	1.005253	-1.421295	-1.127988
29	8	0	2.785144	-2.604386	-1.642574
30	1	0	3.566449	-2.077464	-1.405395
31	1	0	-0.508713	1.385601	-1.726078
32	6	0	-1.767747	2.137465	-0.153438
33	6	0	-2.572694	2.831170	-1.062457
34	6	0	-2.024667	2.254051	1.220156
35	6	0	-3.628094	3.631881	-0.616016
36	1	0	-2.379182	2.748990	-2.127792
37	6	0	-3.068866	3.053045	1.684444
38	1	0	-1.401001	1.726632	1.936024

39	6	0	-3.861218	3.731448	0.754751
40	1	0	-4.251363	4.168804	-1.323169
41	1	0	-3.263776	3.147293	2.747529
42	8	0	0.605923	1.619514	0.035896
43	16	0	1.571236	2.798726	-0.574988
44	8	0	1.436936	3.980263	0.283093
45	8	0	1.312334	2.917085	-2.016220
46	6	0	3.146074	2.021377	-0.287328
47	6	0	3.782936	2.207183	0.941082
48	6	0	3.678201	1.181699	-1.272063
49	6	0	4.973951	1.521714	1.188348
50	1	0	3.355280	2.865677	1.689671
51	6	0	4.868768	0.510589	-1.004656
52	1	0	3.171971	1.055564	-2.222615
53	6	0	5.530148	0.662059	0.228015
54	1	0	5.474614	1.655690	2.143161
55	1	0	5.291821	-0.143298	-1.763002
56	6	0	6.816474	-0.078091	0.491344
57	1	0	7.613333	0.287716	-0.167764
58	1	0	7.147642	0.045860	1.525983
59	1	0	6.698708	-1.149138	0.290930
60	17	0	-5.188066	4.744546	1.333685

Int3

Zero-point correction= 0.435397 (Hartree/Particle)
Thermal correction to Energy= 0.469499
Thermal correction to Enthalpy= 0.470443
Thermal correction to Gibbs Free Energy= 0.363346
Sum of electronic and zero-point Energies= -2611.026078
Sum of electronic and thermal Energies= -2610.991976
Sum of electronic and thermal Enthalpies= -2610.991032
Sum of electronic and thermal Free Energies= -2611.098129

Standard orientation:

Center Number	Atomic Number	Atomic Type	Coordinates (Angstroms) X	Y	Z
1	6	0	-0.629711	-0.518895	-0.485046
2	6	0	-0.401261	0.973855	-0.730406
3	6	0	-1.870385	-1.029653	-1.223817
4	8	0	-1.894200	-0.954037	-2.455194
5	6	0	-2.967639	-1.527279	-0.427764
6	6	0	-3.069480	-1.698075	0.948146
7	16	0	-4.469156	-1.977268	-1.220639
8	6	0	-4.330679	-2.185758	1.353219
9	1	0	-2.266479	-1.479743	1.641839
10	6	0	-5.205205	-2.388656	0.299864

11	1	0	-4.603525	-2.383059	2.383948
12	6	0	-6.610112	-2.897492	0.367620
13	1	0	-6.716045	-3.843246	-0.176722
14	1	0	-6.892558	-3.064792	1.410337
15	1	0	-7.314647	-2.182910	-0.073025
16	1	0	-0.596497	-0.754864	0.577410
17	6	0	3.303510	-1.957919	2.488165
18	6	0	2.840855	-1.548355	1.233960
19	6	0	1.834501	-2.304790	0.630231
20	6	0	1.275832	-3.441792	1.219949
21	6	0	1.752677	-3.826890	2.477086
22	6	0	2.761793	-3.089691	3.106574
23	1	0	4.086264	-1.385514	2.976577
24	1	0	3.248102	-0.667725	0.753758
25	1	0	0.496717	-4.010161	0.723466
26	1	0	1.333723	-4.705924	2.957595
27	1	0	3.127691	-3.399330	4.081007
28	53	0	1.103091	-1.699550	-1.263418
29	1	0	-0.302285	1.165131	-1.801746
30	6	0	-1.568552	1.747644	-0.152404
31	6	0	-2.552182	2.263495	-1.003286
32	6	0	-1.701718	1.892867	1.235259
33	6	0	-3.669244	2.916827	-0.478619
34	1	0	-2.451561	2.157316	-2.079520
35	6	0	-2.808242	2.547201	1.773313
36	1	0	-0.935295	1.505162	1.899349
37	6	0	-3.783551	3.046152	0.905719
38	1	0	-4.431771	3.320334	-1.135988
39	1	0	-2.912961	2.666955	2.846270
40	8	0	0.839587	1.286520	-0.051569
41	16	0	1.737480	2.579903	-0.584489
42	8	0	1.467529	3.706176	0.310184
43	8	0	1.503616	2.717489	-2.026777
44	6	0	3.354009	1.917545	-0.268292
45	6	0	3.933548	2.111264	0.987773
46	6	0	3.993008	1.180759	-1.271717
47	6	0	5.182849	1.543104	1.239515
48	1	0	3.422270	2.689269	1.750177
49	6	0	5.241114	0.626429	-0.997557
50	1	0	3.527730	1.048278	-2.242431
51	6	0	5.853599	0.795086	0.257964
52	1	0	5.643280	1.685174	2.213128
53	1	0	5.749258	0.054305	-1.769068
54	6	0	7.213349	0.204317	0.527482
55	1	0	7.993206	0.812252	0.051570
56	1	0	7.428475	0.164038	1.598938
57	1	0	7.294520	-0.807026	0.115389
58	17	0	-5.191353	3.864298	1.579422

T.S.1

Zero-point correction= 0.433366 (Hartree/Particle)
Thermal correction to Energy= 0.467604
Thermal correction to Enthalpy= 0.468548
Thermal correction to Gibbs Free Energy= 0.360694
Sum of electronic and zero-point Energies= -2611.008794
Sum of electronic and thermal Energies= -2610.974557
Sum of electronic and thermal Enthalpies= -2610.973613
Sum of electronic and thermal Free Energies= -2611.081467

Standard orientation:

Center Number	Atomic Number	Atomic Type	Coordinates (Angstroms) X	Y	Z
1	6	0	-1.069939	0.218905	-0.432330
2	6	0	-0.270333	1.301493	-0.967336
3	6	0	-2.218120	-0.383447	-1.195979
4	8	0	-2.388158	-0.078236	-2.381061
5	6	0	-3.049937	-1.312558	-0.465207
6	6	0	-2.984038	-1.706844	0.868640
7	16	0	-4.355737	-2.138632	-1.293902
8	6	0	-3.969805	-2.654357	1.212727
9	1	0	-2.254304	-1.330825	1.575003
10	6	0	-4.795083	-2.997859	0.154604
11	1	0	-4.083664	-3.080572	2.203209
12	6	0	-5.936674	-3.962733	0.167599
13	1	0	-5.778350	-4.777217	-0.548848
14	1	0	-6.044567	-4.393999	1.166263
15	1	0	-6.877163	-3.467312	-0.100474
16	1	0	-0.854904	-0.098856	0.581965
17	6	0	2.251374	-1.360009	2.909828
18	6	0	2.031965	-1.327420	1.528946
19	6	0	1.150280	-2.254330	0.962795
20	6	0	0.488506	-3.207074	1.744861
21	6	0	0.720317	-3.224666	3.125463
22	6	0	1.597248	-2.304157	3.708871
23	1	0	2.936830	-0.644161	3.354840
24	1	0	2.532697	-0.592057	0.911552
25	1	0	-0.193140	-3.920295	1.293718
26	1	0	0.212282	-3.962391	3.740260
27	1	0	1.772940	-2.324454	4.780520
28	53	0	0.775181	-2.173958	-1.119452
29	1	0	-0.265216	1.415152	-2.050322
30	6	0	-1.324793	2.188400	-0.274923
31	6	0	-2.398920	2.728215	-1.027149
32	6	0	-1.175648	2.523083	1.094529
33	6	0	-3.281307	3.611201	-0.432247

34	1	0	-2.522805	2.452925	-2.068380
35	6	0	-2.061630	3.403598	1.692777
36	1	0	-0.347679	2.114339	1.663435
37	6	0	-3.106767	3.936455	0.924193
38	1	0	-4.095440	4.047100	-1.000378
39	1	0	-1.949011	3.680272	2.734980
40	8	0	1.011354	1.329580	-0.374528
41	16	0	2.131113	2.430749	-0.970888
42	8	0	2.017395	3.652802	-0.174562
43	8	0	1.943526	2.482765	-2.423583
44	6	0	3.579660	1.515791	-0.527980
45	6	0	4.210778	1.790624	0.687157
46	6	0	4.012855	0.490744	-1.377897
47	6	0	5.307448	1.010162	1.055637
48	1	0	3.855024	2.590283	1.327898
49	6	0	5.106062	-0.274327	-0.985456
50	1	0	3.504691	0.295526	-2.316203
51	6	0	5.766522	-0.031703	0.234081
52	1	0	5.811318	1.213429	1.996118
53	1	0	5.454555	-1.074765	-1.632361
54	6	0	6.943153	-0.882441	0.635481
55	1	0	7.343484	-0.584296	1.608046
56	1	0	6.654853	-1.938856	0.689636
57	1	0	7.746657	-0.805657	-0.106511
58	17	0	-4.227157	5.036991	1.674318

Int5 [In electron-withdrawing no Int4 structure]

Zero-point correction= 0.342765 (Hartree/Particle)
Thermal correction to Energy= 0.370110
Thermal correction to Enthalpy= 0.371054
Thermal correction to Gibbs Free Energy= 0.280193
Sum of electronic and zero-point Energies= -2368.071235
Sum of electronic and thermal Energies= -2368.043890
Sum of electronic and thermal Enthalpies= -2368.042946
Sum of electronic and thermal Free Energies= -2368.133807

Standard orientation:

Center Number	Atomic Number	Atomic Type	Coordinates (Angstroms) X	Y	Z
1	6	0	-0.801664	0.166029	-0.170715
2	6	0	0.443215	-0.357847	0.445477
3	6	0	-1.397440	1.306252	0.682235
4	8	0	-1.081982	1.401254	1.870419
5	6	0	-2.343995	2.186326	0.032589
6	6	0	-2.801842	2.180941	-1.276846
7	16	0	-3.116046	3.466552	0.955671

8	6	0	-3.755824	3.191776	-1.533475
9	1	0	-2.467589	1.471852	-2.025229
10	6	0	-4.036565	3.975669	-0.429967
11	1	0	-4.230353	3.349024	-2.495643
12	6	0	-4.986495	5.128728	-0.348735
13	1	0	-4.465148	6.055587	-0.083002
14	1	0	-5.475249	5.272213	-1.315934
15	1	0	-5.760092	4.956990	0.408354
16	1	0	-0.600092	0.481841	-1.195531
17	1	0	0.490414	-0.500470	1.531453
18	6	0	-1.702252	-1.084912	-0.180476
19	6	0	-2.386384	-1.495587	0.973060
20	6	0	-1.806071	-1.838335	-1.358610
21	6	0	-3.194297	-2.632287	0.943918
22	1	0	-2.303988	-0.928456	1.894896
23	6	0	-2.610800	-2.975081	-1.398311
24	1	0	-1.266167	-1.533491	-2.250089
25	6	0	-3.297741	-3.358085	-0.243356
26	1	0	-3.732634	-2.944904	1.831789
27	1	0	-2.702849	-3.551445	-2.312212
28	8	0	1.406694	-0.697395	-0.275793
29	16	0	2.956341	-1.601374	0.581032
30	8	0	2.967185	-2.807963	-0.223746
31	8	0	2.572888	-1.559800	1.983742
32	6	0	4.176320	-0.441566	0.145508
33	6	0	4.813415	-0.578339	-1.097315
34	6	0	4.449816	0.613586	1.033293
35	6	0	5.769863	0.369089	-1.441509
36	1	0	4.575485	-1.399166	-1.764383
37	6	0	5.409392	1.540435	0.655483
38	1	0	3.935806	0.696220	1.984498
39	6	0	6.082143	1.436607	-0.579619
40	1	0	6.283737	0.278754	-2.393228
41	1	0	5.646929	2.360279	1.326533
42	6	0	7.124031	2.453095	-0.949795
43	1	0	7.476053	2.318892	-1.975362
44	1	0	6.728620	3.469107	-0.841095
45	1	0	7.985412	2.370978	-0.275445
46	17	0	-4.315426	-4.792610	-0.287741

Product

Zero-point correction= 0.477394 (Hartree/Particle)
Thermal correction to Energy= 0.515206
Thermal correction to Enthalpy= 0.516150
Thermal correction to Gibbs Free Energy= 0.400975
Sum of electronic and zero-point Energies= -3262.998749
Sum of electronic and thermal Energies= -3262.960937
Sum of electronic and thermal Enthalpies= -3262.959992

Sum of electronic and thermal Free Energies= -3263.075167

Standard orientation:

Center Number	Atomic Number	Atomic Type	Coordinates (Angstroms) X	Y	Z
1	6	0	0.235878	1.199999	0.021177
2	1	0	-0.069012	1.174399	1.069281
3	6	0	-0.632650	0.171393	-0.709831
4	1	0	-0.446874	0.143501	-1.783139
5	6	0	1.718442	0.871694	-0.070683
6	6	0	2.388830	0.894646	-1.301239
7	6	0	2.436231	0.551448	1.088238
8	6	0	3.753138	0.610516	-1.376872
9	1	0	1.848531	1.141098	-2.209518
10	6	0	3.801419	0.266453	1.031820
11	1	0	1.926394	0.518463	2.046664
12	6	0	4.443087	0.302212	-0.205115
13	1	0	4.266738	0.625394	-2.332036
14	1	0	4.350205	0.011564	1.931844
15	8	0	-0.406470	-1.119220	-0.124209
16	16	0	0.128892	-2.406020	-1.033508
17	8	0	0.488686	-1.932921	-2.371561
18	8	0	-0.885483	-3.449198	-0.864518
19	6	0	1.590460	-2.858704	-0.127346
20	6	0	2.832429	-2.743626	-0.748367
21	6	0	1.458831	-3.346125	1.178770
22	6	0	3.971158	-3.129525	-0.038930
23	1	0	2.911146	-2.353939	-1.756196
24	6	0	2.609252	-3.711597	1.869803
25	1	0	0.482597	-3.435374	1.644151
26	6	0	3.880970	-3.611482	1.273485
27	1	0	4.943973	-3.037272	-0.511648
28	1	0	2.522789	-4.083683	2.887144
29	6	0	5.112211	-4.020861	2.040309
30	1	0	5.097777	-5.097094	2.250862
31	1	0	6.025089	-3.794775	1.482606
32	1	0	5.160480	-3.504108	3.005686
33	8	0	-1.975677	0.548586	-0.422681
34	16	0	-3.224368	0.040235	-1.408493
35	8	0	-2.647246	-0.810995	-2.452251
36	8	0	-3.955858	1.258455	-1.754634
37	6	0	-4.184762	-0.926131	-0.266706
38	6	0	-3.898832	-2.285644	-0.121792
39	6	0	-5.185294	-0.296685	0.479716
40	6	0	-4.641885	-3.025760	0.796831

41	1	0	-3.111901	-2.752671	-0.702682
42	6	0	-5.913176	-1.058216	1.391991
43	1	0	-5.391366	0.760079	0.348652
44	6	0	-5.656592	-2.429456	1.563750
45	1	0	-4.428796	-4.084192	0.918346
46	1	0	-6.694083	-0.580984	1.977597
47	6	0	-6.476633	-3.247227	2.528254
48	1	0	-7.398593	-3.594062	2.044047
49	1	0	-5.929542	-4.131389	2.868531
50	1	0	-6.768760	-2.657402	3.402759
51	6	0	-0.022939	2.592740	-0.580833
52	8	0	-0.209419	2.709362	-1.793606
53	6	0	0.021139	3.740767	0.312620
54	6	0	0.214799	3.791974	1.682372
55	16	0	-0.167108	5.358699	-0.345913
56	6	0	0.213265	5.112150	2.196696
57	1	0	0.357394	2.912502	2.299108
58	6	0	0.018075	6.077376	1.229162
59	1	0	0.349834	5.355217	3.244811
60	6	0	-0.042344	7.561582	1.415962
61	1	0	0.732539	8.069831	0.830664
62	1	0	-1.011543	7.964742	1.100308
63	1	0	0.105843	7.807752	2.471027
64	17	0	6.166776	-0.071779	-0.292589

Nitro group (Formation of β, β-ditosyloxy ketone)

Reactant

Zero-point correction= 0.221266 (Hartree/Particle)
Thermal correction to Energy= 0.238376
Thermal correction to Enthalpy= 0.239320
Thermal correction to Gibbs Free Energy= 0.173345
Sum of electronic and zero-point Energies= -1218.499463
Sum of electronic and thermal Energies= -1218.482353
Sum of electronic and thermal Enthalpies= -1218.481409
Sum of electronic and thermal Free Energies= -1218.547384

Standard orientation:

Center Number	Atomic Number	Atomic Type	Coordinates (Angstroms) X	Y	Z
1	6	0	0.625625	-0.318223	-0.000012
2	6	0	-0.493191	-1.071921	-0.000013
3	6	0	1.967322	-0.953094	-0.000013
4	8	0	2.107295	-2.186187	-0.000024
5	6	0	3.135005	-0.068342	-0.000000

6	6	0	3.218998	1.314142	0.000016
7	16	0	4.740776	-0.780699	-0.000006
8	6	0	4.553341	1.793986	0.000023
9	1	0	2.355705	1.969209	0.000024
10	6	0	5.498168	0.788442	0.000012
11	1	0	4.819072	2.845610	0.000036
12	6	0	6.988518	0.930301	0.000016
13	1	0	7.435022	0.460020	-0.883737
14	1	0	7.259833	1.989610	0.000022
15	1	0	7.435018	0.460010	0.883766
16	1	0	0.574085	0.763981	-0.000009
17	1	0	-0.368795	-2.153334	-0.000013
18	6	0	-1.876653	-0.592179	-0.000012
19	6	0	-2.913799	-1.548461	0.000020
20	6	0	-2.223612	0.777214	-0.000039
21	6	0	-4.250560	-1.165589	0.000028
22	1	0	-2.664111	-2.605106	0.000040
23	6	0	-3.552668	1.176313	-0.000033
24	1	0	-1.453190	1.540375	-0.000068
25	6	0	-4.553294	0.197463	0.000002
26	1	0	-5.042920	-1.903802	0.000055
27	1	0	-3.816968	2.226441	-0.000055
28	7	0	-5.955638	0.614957	0.000009
29	8	0	-6.207729	1.825589	-0.000013
30	8	0	-6.828929	-0.260258	0.000037

Int1

Zero-point correction= 0.326650 (Hartree/Particle)
Thermal correction to Energy= 0.353219
Thermal correction to Enthalpy= 0.354163
Thermal correction to Gibbs Free Energy= 0.265983
Sum of electronic and zero-point Energies= -1537.028391
Sum of electronic and thermal Energies= -1537.001822
Sum of electronic and thermal Enthalpies= -1537.000877
Sum of electronic and thermal Free Energies= -1537.089057

Standard orientation:

Center Number	Atomic Number	Atomic Type	Coordinates (Angstroms) X	Y	Z
1	6	0	1.047759	-1.214754	-0.449739
2	6	0	-0.008458	-1.351985	-1.293358
3	6	0	2.438492	-1.129460	-0.985349
4	8	0	2.651698	-1.056308	-2.204766
5	6	0	3.528665	-1.129928	-0.017665
6	6	0	3.496482	-1.212601	1.366624

7	16	0	5.184692	-1.033498	-0.591313
8	6	0	4.783852	-1.197613	1.955612
9	1	0	2.582039	-1.284995	1.943492
10	6	0	5.807951	-1.104131	1.032707
11	1	0	4.964584	-1.255717	3.023484
12	6	0	7.279642	-1.063028	1.301502
13	1	0	7.726620	-0.131855	0.934089
14	1	0	7.460951	-1.132003	2.377435
15	1	0	7.798541	-1.893584	0.809081
16	1	0	0.907900	-1.233159	0.625172
17	1	0	0.198269	-1.328707	-2.361932
18	6	0	-1.409778	-1.502816	-0.907465
19	6	0	-2.397567	-1.306566	-1.894272
20	6	0	-1.810061	-1.802392	0.411513
21	6	0	-3.750041	-1.364985	-1.574238
22	1	0	-2.099366	-1.091001	-2.915930
23	6	0	-3.155560	-1.873977	0.743755
24	1	0	-1.069423	-1.982902	1.182285
25	6	0	-4.107703	-1.640900	-0.253368
26	1	0	-4.510891	-1.197133	-2.326483
27	1	0	-3.467614	-2.094169	1.756764
28	6	0	-3.400228	1.505551	1.223635
29	6	0	-2.404647	1.676608	0.260863
30	6	0	-1.068893	1.599063	0.675720
31	6	0	-0.698070	1.345353	2.002412
32	6	0	-1.714339	1.166869	2.943553
33	6	0	-3.056735	1.248179	2.555661
34	1	0	-4.443286	1.559612	0.927850
35	1	0	-2.661474	1.857789	-0.776815
36	1	0	0.345454	1.290145	2.293236
37	1	0	-1.454105	0.966763	3.978461
38	1	0	-3.839902	1.103869	3.293866
39	53	0	0.443968	1.855510	-0.752071
40	8	0	0.482921	3.848489	-0.726179
41	1	0	-0.172164	4.168649	-1.378446
42	7	0	-5.529526	-1.664407	0.107461
43	8	0	-5.829103	-1.884537	1.285430
44	8	0	-6.361696	-1.452112	-0.780199

Int2

Zero-point correction= 0.459547 (Hartree/Particle)
Thermal correction to Energy= 0.497229
Thermal correction to Enthalpy= 0.498173
Thermal correction to Gibbs Free Energy= 0.383479
Sum of electronic and zero-point Energies= -2431.896387
Sum of electronic and thermal Energies= -2431.858706
Sum of electronic and thermal Enthalpies= -2431.857762
Sum of electronic and thermal Free Energies= -2431.972455

Standard orientation:

Center Number	Atomic Number	Atomic Type	Coordinates (Angstroms) X	Y	Z
1	6	0	-0.721957	-0.406827	-0.354684
2	6	0	-0.726734	1.062946	-0.760204
3	6	0	-1.797788	-1.216535	-0.987820
4	8	0	-2.259058	-0.912302	-2.100392
5	6	0	-2.241782	-2.426511	-0.288556
6	6	0	-1.885771	-2.916145	0.954656
7	16	0	-3.388927	-3.500834	-1.069758
8	6	0	-2.525384	-4.141788	1.277010
9	1	0	-1.184512	-2.418423	1.614049
10	6	0	-3.372576	-4.594713	0.287188
11	1	0	-2.373551	-4.678449	2.207637
12	6	0	-4.207084	-5.838708	0.292558
13	1	0	-3.948674	-6.499583	-0.543060
14	1	0	-4.046676	-6.385972	1.225825
15	1	0	-5.275130	-5.605449	0.209551
16	1	0	-0.667658	-0.514006	0.728057
17	6	0	2.448728	-0.922033	3.023130
18	6	0	2.106560	-0.666406	1.691019
19	6	0	1.778270	-1.744718	0.868438
20	6	0	1.779788	-3.061751	1.330927
21	6	0	2.123190	-3.297932	2.666734
22	6	0	2.456550	-2.233029	3.510938
23	1	0	2.711762	-0.091470	3.672082
24	1	0	2.093584	0.347039	1.310044
25	1	0	1.523471	-3.885335	0.673530
26	1	0	2.125893	-4.317042	3.042740
27	1	0	2.722769	-2.425097	4.546349
28	53	0	1.272415	-1.373711	-1.159028
29	8	0	3.191797	-2.296641	-1.671602
30	1	0	3.890851	-1.661102	-1.443700
31	1	0	-0.638095	1.158957	-1.844418
32	6	0	-1.961524	1.790955	-0.268952
33	6	0	-2.993022	2.100079	-1.164981
34	6	0	-2.095063	2.119941	1.089095
35	6	0	-4.152367	2.730227	-0.717538
36	1	0	-2.891124	1.843234	-2.213940
37	6	0	-3.242959	2.752891	1.552721
38	1	0	-1.292345	1.890306	1.782048
39	6	0	-4.259775	3.047244	0.638266
40	1	0	-4.954037	2.974880	-1.403460
41	1	0	-3.352667	3.015919	2.597574
42	8	0	0.461206	1.633924	-0.122674
43	16	0	1.287800	2.855832	-0.855460

44	8	0	0.921120	4.112402	-0.194763
45	8	0	1.115436	2.726028	-2.308183
46	6	0	2.926978	2.369639	-0.368704
47	6	0	3.423299	2.799514	0.865599
48	6	0	3.654347	1.509436	-1.195431
49	6	0	4.673707	2.342116	1.277666
50	1	0	2.842165	3.469049	1.490884
51	6	0	4.905293	1.068752	-0.764129
52	1	0	3.252680	1.190284	-2.150386
53	6	0	5.430619	1.470097	0.475688
54	1	0	5.067207	2.664768	2.237801
55	1	0	5.480021	0.400309	-1.399583
56	6	0	6.790357	1.002775	0.927621
57	1	0	7.546843	1.769016	0.714771
58	1	0	6.807490	0.818854	2.006755
59	1	0	7.090764	0.086348	0.411455
60	7	0	-5.473461	3.713403	1.117655
61	8	0	-5.559625	3.981388	2.321600
62	8	0	-6.360896	3.978799	0.299294

Int3

Zero-point correction= 0.447680 (Hartree/Particle)
Thermal correction to Energy= 0.482973
Thermal correction to Enthalpy= 0.483918
Thermal correction to Gibbs Free Energy= 0.374490
Sum of electronic and zero-point Energies= -2355.930232
Sum of electronic and thermal Energies= -2355.894939
Sum of electronic and thermal Enthalpies= -2355.893995
Sum of electronic and thermal Free Energies= -2356.003422

Standard orientation:

Center Number	Atomic Number	Atomic Type	Coordinates (Angstroms) X	Y	Z
1	6	0	-0.442247	-0.572895	-0.513738
2	6	0	-0.348340	0.938146	-0.758621
3	6	0	-1.634713	-1.196282	-1.247339
4	8	0	-1.678087	-1.118031	-2.478167
5	6	0	-2.676163	-1.788142	-0.441282
6	6	0	-2.746302	-1.977020	0.935065
7	16	0	-4.149530	-2.344876	-1.219040
8	6	0	-3.961603	-2.562027	1.350988
9	1	0	-1.954506	-1.704400	1.623036
10	6	0	-4.832747	-2.820901	0.306893
11	1	0	-4.205064	-2.787840	2.383191
12	6	0	-6.196729	-3.428935	0.387985
13	1	0	-6.246621	-4.368056	-0.175243

14	1	0	-6.447678	-3.636613	1.431418
15	1	0	-6.956836	-2.756157	-0.025619
16	1	0	-0.386792	-0.797957	0.550326
17	6	0	3.444749	-1.881848	2.552048
18	6	0	2.992651	-1.430009	1.308681
19	6	0	2.105782	-2.245138	0.601428
20	6	0	1.659071	-3.481486	1.075705
21	6	0	2.124925	-3.908260	2.323482
22	6	0	3.012859	-3.113197	3.056569
23	1	0	4.133899	-1.264950	3.120861
24	1	0	3.316623	-0.475415	0.914137
25	1	0	0.972955	-4.092579	0.499485
26	1	0	1.792145	-4.864567	2.715700
27	1	0	3.370506	-3.455568	4.023048
28	53	0	1.394537	-1.581786	-1.280689
29	1	0	-0.223492	1.136798	-1.825931
30	6	0	-1.614775	1.596735	-0.239557
31	6	0	-2.649570	1.905880	-1.131787
32	6	0	-1.776176	1.826411	1.134004
33	6	0	-3.851543	2.427551	-0.658819
34	1	0	-2.520657	1.732852	-2.195580
35	6	0	-2.967661	2.354133	1.621175
36	1	0	-0.968396	1.599489	1.821388
37	6	0	-3.991678	2.637914	0.713938
38	1	0	-4.660241	2.667452	-1.337974
39	1	0	-3.104985	2.537761	2.679558
40	8	0	0.812764	1.377920	-0.023929
41	16	0	1.686039	2.682020	-0.585880
42	8	0	1.357874	3.820471	0.271972
43	8	0	1.472861	2.765195	-2.035252
44	6	0	3.318378	2.087413	-0.223152
45	6	0	3.868569	2.333090	1.037425
46	6	0	4.002860	1.354909	-1.199232
47	6	0	5.134749	1.820897	1.322086
48	1	0	3.323064	2.908540	1.777833
49	6	0	5.267355	0.857632	-0.892457
50	1	0	3.558766	1.182293	-2.173234
51	6	0	5.851096	1.078544	0.368548
52	1	0	5.572565	2.003074	2.299405
53	1	0	5.810848	0.289612	-1.642522
54	6	0	7.228190	0.549399	0.674574
55	1	0	7.992003	1.191357	0.217537
56	1	0	7.417457	0.521042	1.751372
57	1	0	7.364968	-0.457781	0.267266
58	7	0	-5.261375	3.174668	1.223560
59	8	0	-6.169763	3.391436	0.416380
60	8	0	-5.363334	3.379787	2.436900

T.S. 1

Zero-point correction= 0.445628 (Hartree/Particle)
Thermal correction to Energy= 0.481096
Thermal correction to Enthalpy= 0.482040
Thermal correction to Gibbs Free Energy= 0.371211
Sum of electronic and zero-point Energies= -2355.904074
Sum of electronic and thermal Energies= -2355.868606
Sum of electronic and thermal Enthalpies= -2355.867662
Sum of electronic and thermal Free Energies= -2355.978491

Standard orientation:

Center Number	Atomic Number	Atomic Type	Coordinates (Angstroms) X	Y	Z
1	6	0	-1.037800	-0.073104	-0.589731
2	6	0	-0.203623	0.884423	-1.258390
3	6	0	-2.174455	-0.769856	-1.299185
4	8	0	-2.279582	-0.646873	-2.522722
5	6	0	-3.052816	-1.558599	-0.471497
6	6	0	-3.077052	-1.697843	0.914578
7	16	0	-4.323867	-2.510234	-1.215942
8	6	0	-4.101918	-2.554406	1.363037
9	1	0	-2.384226	-1.201263	1.583284
10	6	0	-4.867021	-3.081605	0.334637
11	1	0	-4.285867	-2.789596	2.405321
12	6	0	-6.022907	-4.021403	0.450178
13	1	0	-5.836522	-4.948476	-0.104156
14	1	0	-6.193535	-4.271736	1.500239
15	1	0	-6.938384	-3.574323	0.045167
16	1	0	-0.795624	-0.328170	0.434287
17	6	0	2.587328	-0.800665	2.852798
18	6	0	2.401267	-1.242331	1.538586
19	6	0	1.279188	-2.021745	1.239977
20	6	0	0.344827	-2.362843	2.225062
21	6	0	0.546246	-1.910921	3.534677
22	6	0	1.663961	-1.131130	3.850445
23	1	0	3.457318	-0.194195	3.087455
24	1	0	3.112402	-0.973441	0.766743
25	1	0	-0.522470	-2.967441	1.981309
26	1	0	-0.173018	-2.174819	4.305062
27	1	0	1.814862	-0.784074	4.868544
28	53	0	0.936548	-2.628580	-0.763605
29	1	0	-0.261933	0.940947	-2.344690
30	6	0	-1.271895	1.811013	-0.564276
31	6	0	-2.375752	2.290922	-1.311275

32	6	0	-1.015748	2.291496	0.743671
33	6	0	-3.196822	3.264670	-0.765817
34	1	0	-2.574906	1.900763	-2.303272
35	6	0	-1.840837	3.262153	1.290676
36	1	0	-0.166692	1.915026	1.302721
37	6	0	-2.911692	3.726332	0.523370
38	1	0	-4.038035	3.659984	-1.321214
39	1	0	-1.661486	3.652544	2.284335
40	8	0	1.082626	0.962135	-0.718309
41	16	0	2.113359	2.166286	-1.310970
42	8	0	1.794828	3.398278	-0.588899
43	8	0	2.011357	2.124153	-2.770813
44	6	0	3.616898	1.446299	-0.725166
45	6	0	4.109532	1.835358	0.525077
46	6	0	4.233181	0.453432	-1.492828
47	6	0	5.251647	1.204508	1.010018
48	1	0	3.610516	2.605178	1.103453
49	6	0	5.375469	-0.161631	-0.983968
50	1	0	3.829108	0.162000	-2.456432
51	6	0	5.898653	0.198487	0.269949
52	1	0	5.646363	1.493358	1.980145
53	1	0	5.864291	-0.936753	-1.566911
54	6	0	7.137989	-0.463026	0.812592
55	1	0	7.991638	0.224172	0.757664
56	1	0	7.008820	-0.734553	1.865877
57	1	0	7.393048	-1.363651	0.247492
58	7	0	-3.797341	4.761553	1.110690
59	8	0	-4.777334	5.115069	0.456443
60	8	0	-3.501678	5.205693	2.219454

Int5 [In electron-withdrawing no Int4 structure]

Zero-point correction= 0.354498 (Hartree/Particle)
Thermal correction to Energy= 0.383212
Thermal correction to Enthalpy= 0.384156
Thermal correction to Gibbs Free Energy= 0.289274
Sum of electronic and zero-point Energies= -2112.975907
Sum of electronic and thermal Energies= -2112.947193
Sum of electronic and thermal Enthalpies= -2112.946249
Sum of electronic and thermal Free Energies= -2113.041131

Standard orientation:

Center Number	Atomic Number	Atomic Type	Coordinates (Angstroms) X	Y	Z
1	6	0	0.621162	0.048342	-0.663739
2	6	0	-0.508304	0.846220	-0.059900
3	6	0	0.413574	-1.336696	-0.023874

4	8	0	-0.637025	-1.465527	0.626486
5	6	0	1.371130	-2.390140	-0.223801
6	6	0	2.531056	-2.389692	-0.986566
7	16	0	1.123725	-3.956700	0.533958
8	6	0	3.207852	-3.628499	-0.962496
9	1	0	2.878574	-1.526024	-1.540508
10	6	0	2.578737	-4.583243	-0.183564
11	1	0	4.130834	-3.827475	-1.495350
12	6	0	3.016735	-5.991558	0.064941
13	1	0	2.265430	-6.708116	-0.286216
14	1	0	3.953616	-6.185834	-0.463462
15	1	0	3.175856	-6.176589	1.133373
16	1	0	0.461680	-0.040119	-1.742879
17	1	0	-0.473404	1.096808	1.006580
18	6	0	1.927808	0.774678	-0.377394
19	6	0	2.447401	0.821406	0.925462
20	6	0	2.599878	1.421697	-1.423306
21	6	0	3.639530	1.491533	1.182949
22	1	0	1.930334	0.325461	1.741647
23	6	0	3.795366	2.093813	-1.181821
24	1	0	2.193550	1.393034	-2.429308
25	6	0	4.295898	2.115498	0.120581
26	1	0	4.052774	1.527829	2.183017
27	1	0	4.326874	2.590367	-1.983903
28	8	0	-1.429312	1.277150	-0.775909
29	16	0	-2.880226	2.380634	0.083674
30	8	0	-3.009365	3.377498	-0.960725
31	8	0	-2.292572	2.634660	1.389744
32	6	0	-4.130920	1.176067	0.089144
33	6	0	-4.961719	1.070259	-1.037881
34	6	0	-4.235225	0.320774	1.200839
35	6	0	-5.943884	0.088268	-1.025283
36	1	0	-4.849176	1.737210	-1.885326
37	6	0	-5.226355	-0.647868	1.176036
38	1	0	-3.568121	0.414737	2.050497
39	6	0	-6.094113	-0.780038	0.072166
40	1	0	-6.606544	-0.006374	-1.879584
41	1	0	-5.333437	-1.317221	2.024098
42	6	0	-7.170399	-1.826820	0.087200
43	1	0	-7.644686	-1.934094	-0.891167
44	1	0	-6.765505	-2.796544	0.395798
45	1	0	-7.944894	-1.555454	0.815702
46	7	0	5.557750	2.824346	0.384265
47	8	0	6.125874	3.373989	-0.563210
48	8	0	5.987996	2.836107	1.540723

Product

Zero-point correction= 0.489043 (Hartree/Particle)
Thermal correction to Energy= 0.528182
Thermal correction to Enthalpy= 0.529127
Thermal correction to Gibbs Free Energy= 0.410569
Sum of electronic and zero-point Energies= -3007.908979
Sum of electronic and thermal Energies= -3007.869840
Sum of electronic and thermal Enthalpies= -3007.868895
Sum of electronic and thermal Free Energies= -3007.987454

Standard orientation:

Center Number	Atomic Number	Atomic Type	Coordinates (Angstroms) X	Y	Z
1	6	0	0.448619	1.378645	0.290580
2	1	0	0.273275	1.560632	1.351813
3	6	0	-0.711575	0.496160	-0.192742
4	1	0	-0.645559	0.250563	-1.254024
5	6	0	1.779356	0.661841	0.129892
6	6	0	2.361014	0.480720	-1.134904
7	6	0	2.416262	0.135504	1.262679
8	6	0	3.558176	-0.214694	-1.270627
9	1	0	1.874387	0.880558	-2.017707
10	6	0	3.612418	-0.565003	1.145427
11	1	0	1.964738	0.258995	2.241730
12	6	0	4.167064	-0.728620	-0.124668
13	1	0	4.007521	-0.364684	-2.244371
14	1	0	4.099286	-0.988417	2.014839
15	8	0	-0.701452	-0.678346	0.623490
16	16	0	-0.982180	-2.192345	-0.013885
17	8	0	-1.374582	-2.024513	-1.415212
18	8	0	-1.876221	-2.822427	0.954737
19	6	0	0.618022	-2.963834	0.078766
20	6	0	1.412108	-3.025046	-1.066845
21	6	0	1.032490	-3.523715	1.293019
22	6	0	2.646713	-3.670299	-0.989551
23	1	0	1.072154	-2.588916	-1.999314
24	6	0	2.270234	-4.157358	1.346439
25	1	0	0.396675	-3.473119	2.170484
26	6	0	3.094360	-4.244339	0.209279
27	1	0	3.270971	-3.724656	-1.876500
28	1	0	2.601708	-4.597891	2.282767
29	6	0	4.438004	-4.921978	0.293407
30	1	0	4.355268	-5.898062	0.783711
31	1	0	4.877388	-5.066407	-0.697173

32	1	0	5.135376	-4.317831	0.885862
33	8	0	-1.901351	1.229838	0.088708
34	16	0	-3.229864	1.159725	-0.916021
35	8	0	-2.777804	0.876386	-2.278168
36	8	0	-3.916270	2.411557	-0.596535
37	6	0	-4.154861	-0.219509	-0.281972
38	6	0	-4.411876	-1.309062	-1.113944
39	6	0	-4.638976	-0.161502	1.030208
40	6	0	-5.173023	-2.365197	-0.612388
41	1	0	-4.020011	-1.335195	-2.123283
42	6	0	-5.388393	-1.230383	1.510435
43	1	0	-4.435513	0.697766	1.660941
44	6	0	-5.666674	-2.346862	0.699919
45	1	0	-5.379056	-3.218402	-1.252361
46	1	0	-5.766781	-1.199410	2.528429
47	6	0	-6.455147	-3.507932	1.248814
48	1	0	-7.280640	-3.163956	1.880517
49	1	0	-6.863679	-4.130390	0.447599
50	1	0	-5.811485	-4.143539	1.870386
51	6	0	0.440875	2.705053	-0.489158
52	8	0	0.225485	2.697388	-1.702598
53	6	0	0.738161	3.926376	0.241020
54	6	0	0.988449	4.119037	1.589771
55	16	0	0.829052	5.450677	-0.628211
56	6	0	1.251540	5.470650	1.919895
57	1	0	0.981651	3.319414	2.321052
58	6	0	1.202927	6.320653	0.832810
59	1	0	1.467915	5.818798	2.923977
60	6	0	1.424331	7.801116	0.819954
61	1	0	2.262788	8.074201	0.168897
62	1	0	0.537878	8.334257	0.457347
63	1	0	1.645690	8.150080	1.832264
64	7	0	5.406880	-1.496248	-0.259370
65	8	0	5.991317	-1.851166	0.770454
66	8	0	5.812647	-1.762726	-1.395563

Transition state and product for α, β-ditosyloxy ketone
[Rest all Structures Int1, Int2, Int3 before transition state are the same]

-OCH_3 (T. S. 1)

Zero-point correction= 0.608167 (Hartree/Particle)
Thermal correction to Energy= 0.654769
Thermal correction to Enthalpy= 0.655713
Thermal correction to Gibbs Free Energy= 0.522865
Sum of electronic and zero-point Energies= -3160.775859
Sum of electronic and thermal Energies= -3160.729256
Sum of electronic and thermal Enthalpies= -3160.728312
Sum of electronic and thermal Free Energies= -3160.861160

Standard orientation:

Center Number	Atomic Number	Atomic Type	Coordinates (Angstroms) X	Y	Z
1	6	0	0.126629	0.351882	-0.716469
2	6	0	1.053970	1.528797	-0.873712
3	6	0	-1.043330	0.170699	-1.647488
4	8	0	-1.152286	0.915862	-2.625942
5	6	0	-1.941877	-0.934451	-1.372934
6	6	0	-1.927936	-1.848007	-0.330347
7	16	0	-3.223305	-1.307347	-2.510595
8	6	0	-2.928939	-2.838133	-0.449849
9	1	0	-1.236511	-1.800014	0.499530
10	6	0	-3.721594	-2.684766	-1.570049
11	1	0	-3.086692	-3.629738	0.273565
12	6	0	-4.901888	-3.512470	-1.969055
13	1	0	-4.815395	-3.876371	-2.999079
14	1	0	-4.989925	-4.374311	-1.301911
15	1	0	-5.828625	-2.930428	-1.900287
16	1	0	0.212525	-0.238981	0.180757
17	6	0	1.806186	-3.025247	2.265030
18	6	0	1.980823	-2.278933	1.095779
19	6	0	1.450615	-2.775764	-0.099204
20	6	0	0.764538	-3.992651	-0.158102
21	6	0	0.609228	-4.728375	1.021383
22	6	0	1.124058	-4.246386	2.229489
23	1	0	2.208216	-2.646229	3.199998
24	1	0	2.509906	-1.333508	1.120018
25	1	0	0.350855	-4.355604	-1.092525
26	1	0	0.076065	-5.674200	0.991142
27	1	0	0.992589	-4.820329	3.142108
28	53	0	1.652412	-1.618056	-1.853905
29	1	0	1.452620	1.511239	-1.892515
30	6	0	0.447124	2.899324	-0.627646
31	6	0	0.028059	3.675445	-1.716876
32	6	0	0.339597	3.418708	0.663514
33	6	0	-0.499414	4.945588	-1.515531
34	1	0	0.103893	3.279431	-2.724303
35	6	0	-0.172940	4.697994	0.879849
36	1	0	0.656519	2.824047	1.511177
37	6	0	-0.606606	5.463338	-0.213492
38	1	0	-0.829834	5.552524	-2.352886
39	1	0	-0.248124	5.072297	1.893350
40	8	0	-1.134768	6.715536	-0.114758
41	6	0	-1.320677	7.279941	1.188202
42	1	0	-0.362576	7.405706	1.707313
43	1	0	-1.774844	8.258639	1.023687

44	1	0	-1.993098	6.661928	1.794970
45	8	0	2.121498	1.244241	0.077858
46	16	0	3.654161	1.765470	-0.244826
47	8	0	3.896342	2.984132	0.530486
48	8	0	3.832655	1.783277	-1.702815
49	6	0	4.530014	0.400312	0.481208
50	6	0	4.679498	0.356880	1.871076
51	6	0	4.999119	-0.625027	-0.342654
52	6	0	5.301043	-0.753137	2.439905
53	1	0	4.314902	1.168155	2.492545
54	6	0	5.623932	-1.722549	0.248927
55	1	0	4.876525	-0.566756	-1.417858
56	6	0	5.774597	-1.810178	1.642690
57	1	0	5.420779	-0.799730	3.518825
58	1	0	5.995131	-2.525218	-0.382263
59	6	0	6.398268	-3.027206	2.275756
60	1	0	5.618355	-3.748112	2.554403
61	1	0	7.083373	-3.530855	1.587382
62	1	0	6.945053	-2.767398	3.187542
63	16	0	-2.620929	1.609460	1.300355
64	8	0	-3.034230	2.388503	0.106965
65	8	0	-2.688617	2.343449	2.596986
66	8	0	-1.262600	0.947112	1.136833
67	6	0	-3.802609	0.247948	1.444681
68	6	0	-4.831215	0.117738	0.513829
69	6	0	-5.740196	-0.938811	0.634292
70	6	0	-5.636860	-1.870017	1.675293
71	6	0	-4.596129	-1.711821	2.609210
72	6	0	-3.684493	-0.663046	2.501368
73	1	0	-4.910876	0.827943	-0.301704
74	1	0	-6.533405	-1.042968	-0.101724
75	1	0	-4.499316	-2.421999	3.427538
76	1	0	-2.885310	-0.555711	3.228798
77	6	0	-6.592439	-3.033505	1.784523
78	1	0	-7.394754	-2.963789	1.043933
79	1	0	-6.068788	-3.984870	1.625820
80	1	0	-7.048770	-3.080735	2.780186

Product

Zero-point correction= 0.519322 (Hartree/Particle)
Thermal correction to Energy= 0.558376
Thermal correction to Enthalpy= 0.559320
Thermal correction to Gibbs Free Energy= 0.441800
Sum of electronic and zero-point Energies= -2917.877692
Sum of electronic and thermal Energies= -2917.838638
Sum of electronic and thermal Enthalpies= -2917.837693
Sum of electronic and thermal Free Energies= -2917.955214

Standard orientation:

Center Number	Atomic Number	Atomic Type	Coordinates (Angstroms) X	Y	Z
1	6	0	-0.068929	-0.199116	0.756802
2	6	0	-1.365966	0.583140	1.011464
3	6	0	1.029625	0.032484	1.815590
4	8	0	0.844367	0.803569	2.753873
5	6	0	2.217274	-0.799263	1.687389
6	6	0	2.422005	-1.896596	0.869141
7	16	0	3.614036	-0.516902	2.710376
8	6	0	3.684836	-2.505428	1.063049
9	1	0	1.691209	-2.251277	0.154807
10	6	0	4.457837	-1.872594	2.015165
11	1	0	4.029508	-3.373771	0.513058
12	6	0	5.851579	-2.219445	2.438175
13	1	0	5.912358	-2.406608	3.516319
14	1	0	6.182900	-3.119101	1.912098
15	1	0	6.550420	-1.407426	2.204101
16	1	0	-0.335936	-1.254588	0.844165
17	1	0	-1.602377	0.450226	2.070806
18	6	0	-1.391875	2.045304	0.643369
19	6	0	-1.096978	3.017980	1.611249
20	6	0	-1.690883	2.457959	-0.656182
21	6	0	-1.084815	4.367878	1.279667
22	1	0	-0.860379	2.710817	2.624364
23	6	0	-1.687847	3.811625	-1.005113
24	1	0	-1.933202	1.720244	-1.412108
25	6	0	-1.375860	4.773113	-0.034184
26	1	0	-0.854982	5.124025	2.024436
27	1	0	-1.921338	4.095645	-2.024070
28	8	0	-1.338307	6.118325	-0.263245
29	6	0	-1.621754	6.594179	-1.582614
30	1	0	-2.640187	6.327880	-1.891207
31	1	0	-1.531142	7.680679	-1.531451
32	1	0	-0.900825	6.201970	-2.310399
33	8	0	-2.341482	-0.176932	0.218918
34	16	0	-3.918923	-0.193213	0.674094
35	8	0	-4.644638	0.810815	-0.110934
36	8	0	-3.983926	-0.142498	2.141149
37	6	0	-4.316292	-1.823105	0.082697
38	6	0	-4.464750	-2.023019	-1.294247
39	6	0	-4.419986	-2.875127	0.993330
40	6	0	-4.707551	-3.313343	-1.758272
41	1	0	-4.387574	-1.190629	-1.986171
42	6	0	-4.672257	-4.158507	0.505869
43	1	0	-4.298345	-2.698304	2.055577
44	6	0	-4.809635	-4.399573	-0.869673

45	1	0	-4.820620	-3.481910	-2.825868
46	1	0	-4.754097	-4.984034	1.207291
47	6	0	-5.032819	-5.795227	-1.393843
48	1	0	-4.086110	-6.223438	-1.747967
49	1	0	-5.427211	-6.457131	-0.617390
50	1	0	-5.727447	-5.796028	-2.240181
51	16	0	1.382556	1.052184	-1.215690
52	8	0	1.671038	2.033949	-0.168111
53	8	0	0.763699	1.454809	-2.479937
54	8	0	0.368307	-0.111542	-0.623564
55	6	0	2.855885	0.115522	-1.573253
56	6	0	4.051370	0.471506	-0.948954
57	6	0	5.202137	-0.260725	-1.242777
58	6	0	5.168022	-1.341137	-2.137363
59	6	0	3.946376	-1.667191	-2.753520
60	6	0	2.787315	-0.943316	-2.483521
61	1	0	4.079428	1.292904	-0.242178
62	1	0	6.137557	0.006664	-0.759034
63	1	0	3.903575	-2.497255	-3.453669
64	1	0	1.849985	-1.204042	-2.963896
65	6	0	6.403995	-2.162637	-2.402357
66	1	0	7.315121	-1.576834	-2.247292
67	1	0	6.443012	-3.021119	-1.718763
68	1	0	6.411168	-2.556670	-3.423543

Transition state and product for α, β-ditosyloxy ketone
-SCH_3 Group (T. S. 1)

Zero-point correction= 0.603662 (Hartree/Particle)
Thermal correction to Energy= 0.651309
Thermal correction to Enthalpy= 0.652254
Thermal correction to Gibbs Free Energy= 0.515419
Sum of electronic and zero-point Energies= -3483.756576
Sum of electronic and thermal Energies= -3483.708928
Sum of electronic and thermal Enthalpies= -3483.707984
Sum of electronic and thermal Free Energies= -3483.844818
Input orientation:

Center Number	Atomic Number	Atomic Type	Coordinates (Angstroms) X	Y	Z
1	6	0	0.103854	0.346219	-0.670619
2	6	0	0.951637	1.588060	-0.802697
3	6	0	-1.034427	0.106044	-1.628285
4	8	0	-1.151540	0.844376	-2.610832
5	6	0	-1.892892	-1.034840	-1.375213
6	6	0	-1.857720	-1.960986	-0.345217
7	16	0	-3.155296	-1.430704	-2.526460

8	6	0	-2.824140	-2.982725	-0.487018
9	1	0	-1.172896	-1.902117	0.489401
10	6	0	-3.613195	-2.839148	-1.611046
11	1	0	-2.960030	-3.790070	0.223346
12	6	0	-4.755329	-3.705301	-2.039335
13	1	0	-4.620239	-4.078944	-3.060821
14	1	0	-4.841585	-4.561716	-1.364948
15	1	0	-5.701101	-3.151128	-2.013133
16	1	0	0.229836	-0.259739	0.211452
17	6	0	2.029483	-3.134411	2.191428
18	6	0	2.167749	-2.308999	1.071277
19	6	0	1.627297	-2.738676	-0.144682
20	6	0	0.962216	-3.962424	-0.271392
21	6	0	0.842381	-4.776764	0.859358
22	6	0	1.370739	-4.364429	2.087347
23	1	0	2.441020	-2.809454	3.142541
24	1	0	2.679972	-1.357361	1.150505
25	1	0	0.538400	-4.270888	-1.220744
26	1	0	0.327346	-5.729396	0.775785
27	1	0	1.268647	-5.000038	2.962061
28	53	0	1.773910	-1.472637	-1.826294
29	1	0	1.356559	1.613353	-1.818992
30	6	0	0.259690	2.916624	-0.545571
31	6	0	-0.192318	3.680706	-1.627620
32	6	0	0.107094	3.410135	0.751504
33	6	0	-0.807039	4.911385	-1.413975
34	1	0	-0.080420	3.306156	-2.639842
35	6	0	-0.495989	4.646768	0.974539
36	1	0	0.452479	2.826501	1.595614
37	6	0	-0.970846	5.405775	-0.106770
38	1	0	-1.161514	5.485391	-2.266090
39	1	0	-0.604048	4.995240	1.994540
40	8	0	2.029899	1.365304	0.150710
41	16	0	3.532139	1.969221	-0.171984
42	8	0	3.707640	3.191753	0.614795
43	8	0	3.704967	2.011235	-1.630092
44	6	0	4.483696	0.645304	0.535499
45	6	0	4.599601	0.566546	1.926931
46	6	0	5.048853	-0.314757	-0.306658
47	6	0	5.283188	-0.515638	2.478006
48	1	0	4.161453	1.328175	2.563677
49	6	0	5.735443	-1.384228	0.267861
50	1	0	4.951997	-0.229151	-1.382665
51	6	0	5.852703	-1.508970	1.661902
52	1	0	5.376073	-0.590656	3.557960
53	1	0	6.181131	-2.136007	-0.377611
54	6	0	6.542699	-2.701015	2.273464
55	1	0	5.811227	-3.492241	2.484494
56	1	0	7.294594	-3.119158	1.597516

57	1	0	7.027157	-2.441038	3.219682
58	16	0	-2.706192	1.427472	1.286837
59	8	0	-3.169067	2.137061	0.069654
60	8	0	-2.815888	2.196238	2.559430
61	8	0	-1.306531	0.847052	1.139869
62	6	0	-3.794319	-0.002180	1.485932
63	6	0	-4.872492	-0.185715	0.621844
64	6	0	-5.720146	-1.283713	0.802227
65	6	0	-5.504921	-2.204982	1.835949
66	6	0	-4.414404	-1.994328	2.699369
67	6	0	-3.563607	-0.902742	2.532169
68	1	0	-5.039662	0.518159	-0.185986
69	1	0	-6.556595	-1.426063	0.122501
70	1	0	-4.232384	-2.694848	3.511275
71	1	0	-2.726423	-0.753257	3.207490
72	6	0	-6.392582	-3.414439	2.002410
73	1	0	-7.342949	-3.290480	1.474141
74	1	0	-5.904055	-4.311954	1.600432
75	1	0	-6.608132	-3.609367	3.058617
76	16	0	-1.768659	6.990469	0.062481
77	6	0	-1.970596	7.182424	1.865570
78	1	0	-2.559228	6.362100	2.284603
79	1	0	-1.005469	7.256682	2.373610
80	1	0	-2.516178	8.120086	2.002252

Product

Zero-point correction= 0.516022 (Hartree/Particle)
Thermal correction to Energy= 0.554808
Thermal correction to Enthalpy= 0.555752
Thermal correction to Gibbs Free Energy= 0.440966
Sum of electronic and zero-point Energies= -3240.864510
Sum of electronic and thermal Energies= -3240.825725
Sum of electronic and thermal Enthalpies= -3240.824781
Sum of electronic and thermal Free Energies= -3240.939566

Standard orientation:

Center Number	Atomic Number	Atomic Type	Coordinates (Angstroms) X	Y	Z
1	6	0	-0.149952	-0.673145	-0.067247
2	6	0	-1.516297	-1.343057	0.177046
3	6	0	0.596345	-0.312670	1.238978
4	8	0	0.069422	-0.496714	2.333476
5	6	0	1.970628	0.137209	1.083518
6	6	0	2.761320	0.143832	-0.052836
7	16	0	2.875647	0.710671	2.472370
8	6	0	4.077194	0.603824	0.188319

9	1	0	2.404532	-0.165065	-1.027563
10	6	0	4.300032	0.961541	1.502947
11	1	0	4.839882	0.689087	-0.577583
12	6	0	5.554896	1.522798	2.094947
13	1	0	5.908441	0.921237	2.939802
14	1	0	6.340809	1.547684	1.335110
15	1	0	5.397577	2.544628	2.460052
16	1	0	0.476213	-1.417630	-0.565135
17	1	0	-1.404682	-1.978899	1.057628
18	6	0	-2.704652	-0.438612	0.373164
19	6	0	-3.093286	-0.075754	1.669969
20	6	0	-3.417800	0.072122	-0.713489
21	6	0	-4.162423	0.789892	1.873766
22	1	0	-2.539813	-0.456451	2.522129
23	6	0	-4.496252	0.938370	-0.519437
24	1	0	-3.135162	-0.201110	-1.724031
25	6	0	-4.873939	1.312977	0.777603
26	1	0	-4.441704	1.062819	2.888106
27	1	0	-5.021313	1.318327	-1.387627
28	6	0	-6.827171	2.930454	-0.499601
29	1	0	-7.222357	2.080306	-1.061712
30	1	0	-7.638988	3.637413	-0.308777
31	1	0	-6.043929	3.434154	-1.072215
32	8	0	-1.766931	-2.190501	-1.000721
33	16	0	-1.590572	-3.812903	-0.827167
34	8	0	-1.945523	-4.333981	-2.147277
35	8	0	-2.324062	-4.241178	0.368892
36	6	0	0.157977	-3.998185	-0.535102
37	6	0	1.035776	-3.937341	-1.622716
38	6	0	0.626180	-4.087109	0.778715
39	6	0	2.407114	-3.960492	-1.377568
40	1	0	0.654170	-3.869905	-2.636176
41	6	0	2.003018	-4.105744	1.000360
42	1	0	-0.068466	-4.135623	1.610163
43	6	0	2.912218	-4.033925	-0.067068
44	1	0	3.096831	-3.918704	-2.216068
45	1	0	2.374083	-4.171288	2.019194
46	6	0	4.396939	-4.000838	0.187702
47	1	0	4.738506	-2.963228	0.296064
48	1	0	4.655998	-4.529764	1.109698
49	1	0	4.954864	-4.446736	-0.641201
50	16	0	-0.518409	1.975097	-0.728670
51	8	0	-0.895886	2.111008	0.679058
52	8	0	-1.423270	2.385375	-1.802361
53	8	0	-0.203068	0.379658	-1.063426
54	6	0	1.069790	2.742776	-0.990470
55	6	0	1.693314	3.387282	0.079187
56	6	0	2.935588	3.982582	-0.132311
57	6	0	3.562101	3.933617	-1.387572

58	6	0	2.899449	3.287582	-2.445111
59	6	0	1.651854	2.694124	-2.259359
60	1	0	1.223249	3.413200	1.055322
61	1	0	3.430025	4.483389	0.695449
62	1	0	3.364708	3.249115	-3.426133
63	1	0	1.148787	2.198497	-3.083315
64	6	0	4.934079	4.527638	-1.578768
65	1	0	5.045881	5.460027	-1.016259
66	1	0	5.701423	3.832620	-1.213403
67	1	0	5.142446	4.728785	-2.633512
68	16	0	-6.222396	2.422966	1.145683

Transition state and product for α, β-ditosyloxy ketone
Chloride Group (T.S.1)

Zero-point correction= 0.565231 (Hartree/Particle)
Thermal correction to Energy= 0.610845
Thermal correction to Enthalpy= 0.611789
Thermal correction to Gibbs Free Energy= 0.478119
Sum of electronic and zero-point Energies= -3505.879578
Sum of electronic and thermal Energies= -3505.833964
Sum of electronic and thermal Enthalpies= -3505.833020
Sum of electronic and thermal Free Energies= -3505.966689

Input orientation:

Center Number	Atomic Number	Atomic Type	Coordinates (Angstroms) X	Y	Z
1	6	0	0.131769	0.303663	-0.671734
2	6	0	0.957967	1.561377	-0.815504
3	6	0	-1.005256	0.039521	-1.624636
4	8	0	-1.116699	0.751625	-2.626768
5	6	0	-1.864543	-1.093923	-1.345347
6	6	0	-1.844523	-1.982231	-0.282240
7	16	0	-3.109541	-1.532383	-2.500210
8	6	0	-2.809421	-3.008303	-0.400658
9	1	0	-1.172783	-1.892310	0.560354
10	6	0	-3.581636	-2.906087	-1.540683
11	1	0	-2.956444	-3.789185	0.336615
12	6	0	-4.719855	-3.785790	-1.951222
13	1	0	-4.582196	-4.182975	-2.963382
14	1	0	-4.805399	-4.626028	-1.256688
15	1	0	-5.666939	-3.233318	-1.939026
16	1	0	0.255156	-0.275293	0.228598
17	6	0	2.084214	-3.154920	2.227049
18	6	0	2.216149	-2.330992	1.105162
19	6	0	1.654020	-2.755252	-0.102853

20	6	0	0.974181	-3.971620	-0.221249
21	6	0	0.861379	-4.784583	0.911217
22	6	0	1.411008	-4.377828	2.131689
23	1	0	2.511975	-2.834501	3.172513
24	1	0	2.739471	-1.384937	1.177189
25	1	0	0.534159	-4.275198	-1.164737
26	1	0	0.335067	-5.731612	0.835052
27	1	0	1.314220	-5.012434	3.007721
28	53	0	1.794201	-1.491995	-1.787242
29	1	0	1.377628	1.577522	-1.826043
30	6	0	0.243401	2.889007	-0.593018
31	6	0	-0.266854	3.586677	-1.690981
32	6	0	0.150264	3.442560	0.686816
33	6	0	-0.878406	4.829762	-1.518546
34	1	0	-0.199138	3.159252	-2.685323
35	6	0	-0.451073	4.685860	0.875079
36	1	0	0.542416	2.904957	1.540597
37	6	0	-0.962335	5.362223	-0.232748
38	1	0	-1.277320	5.371030	-2.369659
39	1	0	-0.531277	5.112356	1.868909
40	8	0	2.024754	1.378204	0.154327
41	16	0	3.526880	1.990361	-0.164402
42	8	0	3.681488	3.225925	0.605658
43	8	0	3.710826	2.011925	-1.621300
44	6	0	4.483383	0.686582	0.571485
45	6	0	4.590551	0.632955	1.964827
46	6	0	5.062760	-0.282142	-0.250951
47	6	0	5.282051	-0.432349	2.538402
48	1	0	4.140708	1.400786	2.585705
49	6	0	5.756930	-1.334137	0.346193
50	1	0	4.971455	-0.215991	-1.328879
51	6	0	5.867608	-1.433056	1.742852
52	1	0	5.369126	-0.487674	3.620007
53	1	0	6.214561	-2.092236	-0.283305
54	6	0	6.568610	-2.605533	2.379145
55	1	0	5.844564	-3.398528	2.608284
56	1	0	7.323681	-3.031316	1.711619
57	1	0	7.051493	-2.320911	3.319076
58	16	0	-2.677410	1.478757	1.198062
59	8	0	-3.073259	2.162435	-0.056697
60	8	0	-2.812905	2.286569	2.443072
61	8	0	-1.287067	0.862351	1.117110
62	6	0	-3.808991	0.083089	1.395133
63	6	0	-4.841169	-0.112252	0.479474
64	6	0	-5.720311	-1.186634	0.651841
65	6	0	-5.582805	-2.071537	1.729108
66	6	0	-4.538571	-1.848112	2.645424
67	6	0	-3.656586	-0.780661	2.486181
68	1	0	-4.947857	0.563128	-0.362114

69	1	0	-6.518881	-1.340127	-0.069648
70	1	0	-4.416937	-2.521067	3.491276
71	1	0	-2.854530	-0.622324	3.200992
72	6	0	-6.503866	-3.256259	1.892113
73	1	0	-7.370183	-3.185445	1.227356
74	1	0	-5.978435	-4.191343	1.657974
75	1	0	-6.867072	-3.340077	2.922615
76	17	0	-1.729228	6.937332	-0.001116

Product

Zero-point correction= 0.477251 (Hartree/Particle)
Thermal correction to Energy= 0.514864
Thermal correction to Enthalpy= 0.515808
Thermal correction to Gibbs Free Energy= 0.402175
Sum of electronic and zero-point Energies= -3262.988915
Sum of electronic and thermal Energies= -3262.951302
Sum of electronic and thermal Enthalpies= -3262.950358
Sum of electronic and thermal Free Energies= -3263.063990

Standard orientation:

Center Number	Atomic Number	Atomic Type	Coordinates (Angstroms) X	Y	Z
1	6	0	0.574216	-1.286906	0.062128
2	6	0	-0.927590	-1.311404	0.384168
3	6	0	1.463874	-1.172127	1.323212
4	8	0	0.955530	-1.080518	2.437726
5	6	0	2.898187	-1.276002	1.106966
6	6	0	3.579021	-1.671693	-0.031459
7	16	0	4.018133	-0.941651	2.415182
8	6	0	4.982538	-1.706172	0.144058
9	1	0	3.084935	-1.918937	-0.963001
10	6	0	5.385535	-1.326976	1.408580
11	1	0	5.684250	-1.983770	-0.634317
12	6	0	6.785864	-1.206933	1.923063
13	1	0	6.936432	-1.802835	2.830122
14	1	0	7.489199	-1.553813	1.161226
15	1	0	7.029207	-0.166163	2.167521
16	1	0	0.801384	-2.258858	-0.387999
17	1	0	-1.043541	-2.002102	1.222749
18	6	0	-1.605163	0.002346	0.698390
19	6	0	-1.832661	0.366362	2.029599
20	6	0	-2.050531	0.840864	-0.328376
21	6	0	-2.494270	1.555056	2.339972
22	1	0	-1.489553	-0.278958	2.830760
23	6	0	-2.715937	2.030854	-0.037461
24	1	0	-1.895434	0.561440	-1.363111

25	6	0	-2.931438	2.371090	1.296953
26	1	0	-2.675354	1.833055	3.372563
27	1	0	-3.067182	2.674886	-0.836060
28	8	0	-1.519890	-1.926568	-0.809018
29	16	0	-2.904165	-2.806696	-0.659957
30	8	0	-2.882613	-3.482383	0.643278
31	8	0	-2.935018	-3.580050	-1.902499
32	6	0	-4.205481	-1.593334	-0.678057
33	6	0	-4.752400	-1.158582	0.531598
34	6	0	-4.597849	-1.044409	-1.901192
35	6	0	-5.703998	-0.141798	0.507055
36	1	0	-4.430345	-1.595151	1.469924
37	6	0	-5.552747	-0.029116	-1.901993
38	1	0	-4.163438	-1.397845	-2.830237
39	6	0	-6.111043	0.444618	-0.702983
40	1	0	-6.128256	0.209954	1.443178
41	1	0	-5.864138	0.405671	-2.847618
42	6	0	-7.095027	1.585767	-0.705592
43	1	0	-6.576712	2.529271	-0.491040
44	1	0	-7.587748	1.690521	-1.676519
45	1	0	-7.861883	1.453711	0.064292
46	16	0	1.275240	1.237382	-0.794549
47	8	0	1.075381	1.595207	0.611105
48	8	0	0.531344	1.911736	-1.859425
49	8	0	0.908433	-0.367818	-1.011102
50	6	0	3.014288	1.298926	-1.178790
51	6	0	3.903164	1.743212	-0.199625
52	6	0	5.260663	1.811648	-0.511516
53	6	0	5.736072	1.434566	-1.776561
54	6	0	4.809888	1.000071	-2.741543
55	6	0	3.448174	0.934055	-2.456405
56	1	0	3.543621	2.020727	0.784347
57	1	0	5.960284	2.156051	0.244776
58	1	0	5.159930	0.712301	-3.729079
59	1	0	2.742197	0.599828	-3.209618
60	6	0	7.211616	1.454959	-2.083312
61	1	0	7.736249	2.200316	-1.478092
62	1	0	7.657968	0.476610	-1.861976
63	1	0	7.397763	1.668669	-3.140277
64	17	0	-3.799664	3.864139	1.675347

Transition state and product for α, β-ditosyloxy ketone

T.S. 1 (Nitro group)

Zero-point correction= 0.578137 (Hartree/Particle)
Thermal correction to Energy= 0.624749
Thermal correction to Enthalpy= 0.625693
Thermal correction to Gibbs Free Energy= 0.492090

Sum of electronic and zero-point Energies= -3250.785765
Sum of electronic and thermal Energies= -3250.739153
Sum of electronic and thermal Enthalpies= -3250.738209
Sum of electronic and thermal Free Energies= -3250.871812

Standard orientation:

Center Number	Atomic Number	Atomic Type	Coordinates (Angstroms) X	Y	Z
1	6	0	0.156510	0.194052	-0.676414
2	6	0	0.852848	1.533023	-0.797784
3	6	0	-0.977712	-0.170632	-1.599102
4	8	0	-1.187078	0.527301	-2.595681
5	6	0	-1.717684	-1.379550	-1.292251
6	6	0	-1.601126	-2.239694	-0.211485
7	16	0	-2.938473	-1.950757	-2.414147
8	6	0	-2.476294	-3.346647	-0.289360
9	1	0	-0.928908	-2.068948	0.618775
10	6	0	-3.274555	-3.336996	-1.416057
11	1	0	-2.543571	-4.119339	0.468043
12	6	0	-4.345847	-4.315395	-1.780847
13	1	0	-4.231990	-4.682261	-2.807006
14	1	0	-4.313285	-5.170617	-1.100170
15	1	0	-5.337336	-3.853476	-1.702242
16	1	0	0.350150	-0.381758	0.214072
17	6	0	2.445863	-3.133837	2.099483
18	6	0	2.500937	-2.274633	0.997980
19	6	0	1.941528	-2.703733	-0.209685
20	6	0	1.339709	-3.958261	-0.348146
21	6	0	1.304091	-4.805801	0.763788
22	6	0	1.850765	-4.395036	1.984136
23	1	0	2.871558	-2.810427	3.044861
24	1	0	2.964959	-1.299919	1.085063
25	1	0	0.899621	-4.264995	-1.290537
26	1	0	0.838786	-5.782896	0.672099
27	1	0	1.812892	-5.056607	2.844659
28	53	0	1.952337	-1.386573	-1.857902
29	1	0	1.248255	1.625157	-1.813331
30	6	0	0.010706	2.775282	-0.524414
31	6	0	-0.656653	3.394230	-1.586143
32	6	0	-0.044522	3.325652	0.760384
33	6	0	-1.395329	4.553207	-1.369614
34	1	0	-0.612798	2.963122	-2.579179
35	6	0	-0.772520	4.486864	0.992261
36	1	0	0.472026	2.844025	1.579884
37	6	0	-1.441175	5.083351	-0.079267
38	1	0	-1.920885	5.038385	-2.182524
39	1	0	-0.833475	4.914255	1.985036

40	8	0	1.947347	1.436646	0.148573
41	16	0	3.391092	2.172652	-0.197280
42	8	0	3.449175	3.416485	0.572299
43	8	0	3.542898	2.210211	-1.657254
44	6	0	4.466678	0.954334	0.518244
45	6	0	4.591772	0.900255	1.910633
46	6	0	5.122899	0.047738	-0.317361
47	6	0	5.381233	-0.102683	2.468792
48	1	0	4.082043	1.620304	2.542450
49	6	0	5.913525	-0.942951	0.265250
50	1	0	5.019036	0.114448	-1.394142
51	6	0	6.046549	-1.041323	1.659594
52	1	0	5.484157	-0.157234	3.549032
53	1	0	6.430876	-1.652390	-0.374564
54	6	0	6.858170	-2.147003	2.283616
55	1	0	6.200152	-2.968762	2.595296
56	1	0	7.588562	-2.555664	1.579345
57	1	0	7.388103	-1.797257	3.175316
58	16	0	-2.712615	1.183758	1.225142
59	8	0	-3.151316	1.870755	-0.014176
60	8	0	-2.878413	1.960121	2.485779
61	8	0	-1.291308	0.643835	1.120733
62	6	0	-3.765311	-0.272548	1.404200
63	6	0	-4.736827	-0.553736	0.445643
64	6	0	-5.552035	-1.679391	0.601750
65	6	0	-5.410201	-2.531147	1.704876
66	6	0	-4.428980	-2.220771	2.664320
67	6	0	-3.610616	-1.101503	2.521666
68	1	0	-4.847621	0.096478	-0.414909
69	1	0	-6.304533	-1.898244	-0.151621
70	1	0	-4.307065	-2.865500	3.531756
71	1	0	-2.859062	-0.874066	3.271735
72	6	0	-6.258237	-3.771093	1.851028
73	1	0	-7.111800	-3.757035	1.166534
74	1	0	-5.669167	-4.671201	1.631810
75	1	0	-6.638748	-3.875827	2.873232
76	7	0	-2.213457	6.305063	0.157926
77	8	0	-2.223780	6.782879	1.297993
78	8	0	-2.822481	6.810392	-0.792411

Product (Nitro group)

Zero-point correction= 0.489474 (Hartree/Particle)
Thermal correction to Energy= 0.528247
Thermal correction to Enthalpy= 0.529191
Thermal correction to Gibbs Free Energy= 0.413894
Sum of electronic and zero-point Energies= -3007.896202
Sum of electronic and thermal Energies= -3007.857429
Sum of electronic and thermal Enthalpies= -3007.856485

Sum of electronic and thermal Free Energies= -3007.971783

Standard orientation:

Center Number	Atomic Number	Atomic Type	Coordinates (Angstroms) X	Y	Z
1	6	0	0.667420	-1.341940	0.091626
2	6	0	-0.839669	-1.372624	0.397068
3	6	0	1.541677	-1.144314	1.353065
4	8	0	1.018585	-1.001784	2.455851
5	6	0	2.978280	-1.235755	1.156242
6	6	0	3.676853	-1.669904	0.042261
7	16	0	4.079234	-0.826438	2.459309
8	6	0	5.078452	-1.673322	0.232525
9	1	0	3.195996	-1.964438	-0.882529
10	6	0	5.462422	-1.232734	1.483360
11	1	0	5.792639	-1.973801	-0.525698
12	6	0	6.855235	-1.069222	2.006036
13	1	0	7.007436	-1.631786	2.933911
14	1	0	7.572234	-1.428194	1.262775
15	1	0	7.076989	-0.016599	2.218000
16	1	0	0.910610	-2.335534	-0.298851
17	1	0	-0.957953	-1.992983	1.288724
18	6	0	-1.534390	-0.042970	0.596563
19	6	0	-1.707698	0.462378	1.891713
20	6	0	-2.032242	0.668113	-0.501634
21	6	0	-2.372649	1.666610	2.095309
22	1	0	-1.316196	-0.087098	2.739578
23	6	0	-2.705868	1.870110	-0.316775
24	1	0	-1.910634	0.274380	-1.502544
25	6	0	-2.868124	2.351754	0.983092
26	1	0	-2.517366	2.062048	3.092887
27	1	0	-3.100003	2.422189	-1.160395
28	8	0	-1.410178	-2.080797	-0.747702
29	16	0	-2.753536	-3.016714	-0.530620
30	8	0	-2.672237	-3.630974	0.799803
31	8	0	-2.771692	-3.841954	-1.738418
32	6	0	-4.100430	-1.856097	-0.569617
33	6	0	-4.631457	-1.384220	0.633314
34	6	0	-4.539637	-1.375590	-1.806046
35	6	0	-5.613622	-0.397051	0.587633
36	1	0	-4.277463	-1.771760	1.581832
37	6	0	-5.522567	-0.388332	-1.827337
38	1	0	-4.117889	-1.758251	-2.729302
39	6	0	-6.064369	0.124552	-0.636414
40	1	0	-6.028750	-0.018735	1.517512
41	1	0	-5.869598	-0.005531	-2.782893
42	6	0	-7.077870	1.238893	-0.666244

43	1	0	-6.575185	2.207032	-0.543915
44	1	0	-7.619487	1.264780	-1.616230
45	1	0	-7.802476	1.142202	0.148287
46	16	0	1.318827	1.144063	-0.898002
47	8	0	1.069137	1.571048	0.480520
48	8	0	0.581419	1.732877	-2.016221
49	8	0	0.998571	-0.482385	-1.029049
50	6	0	3.064304	1.236285	-1.240263
51	6	0	3.914967	1.745954	-0.258390
52	6	0	5.277467	1.837957	-0.538670
53	6	0	5.795714	1.421097	-1.774401
54	6	0	4.907492	0.920656	-2.742710
55	6	0	3.540698	0.829582	-2.489634
56	1	0	3.522497	2.056853	0.702742
57	1	0	5.948076	2.232666	0.219344
58	1	0	5.291033	0.601275	-3.707754
59	1	0	2.863740	0.445442	-3.245629
60	6	0	7.277991	1.470791	-2.042200
61	1	0	7.755714	2.290499	-1.496992
62	1	0	7.753458	0.536998	-1.714426
63	1	0	7.488766	1.590508	-3.109149
64	7	0	-3.598169	3.605505	1.188025
65	8	0	-4.094559	4.164168	0.203286
66	8	0	-3.691756	4.049904	2.337768

--------------------End of File-------------------------------

----
